# Ultrasound Neuromodulation: A Review of Results, Mechanisms and Safety

**DOI:** 10.1016/j.ultrasmedbio.2018.12.015

**Published:** 2019-07

**Authors:** Joseph Blackmore, Shamit Shrivastava, Jerome Sallet, Chris R. Butler, Robin O. Cleveland

**Affiliations:** ⁎ Institute of Biomedical Engineering, Department of Engineering Science, University of Oxford, Roosevelt Drive, Oxford, UK; † Wellcome Centre for Integrative Nueroimaging, Department of Experimental Psychology, University of Oxford, Oxford, UK; ‡ Nuffield Department of Clinical Neurosciences, John Radcliffe Hospital, Oxford, UK

**Keywords:** Ultrasound, Neuromodulation, Brain stimulation, Non-invasive, Mechanisms, Safety

## Abstract

Ultrasonic neuromodulation is a rapidly growing field, in which low-intensity ultrasound (US) is delivered to nervous system tissue, resulting in transient modulation of neural activity. This review summarizes the findings in the central and peripheral nervous systems from mechanistic studies in cell culture to cognitive behavioral studies in humans. The mechanisms by which US mechanically interacts with neurons and could affect firing are presented. An in-depth safety assessment of current studies shows that parameters for the human studies fall within the safety envelope for US imaging. Challenges associated with accurately targeting US and monitoring the response are described. In conclusion, the literature supports the use of US as a safe, non-invasive brain stimulation modality with improved spatial localization and depth targeting compared with alternative methods. US neurostimulation has the potential to be used both as a scientific instrument to investigate brain function and as a therapeutic modality to modulate brain activity.

## Introduction

Brain stimulation techniques are vital tools to probe neurologic processes from a cellular scale all the way up to a systems level. Approaches such as the local injection of pharmacologic agents, like muscimol (Amiez et al. 2006), micro-stimulation (Histed et al. 2009) and optogenetics (Boyden 2011) allow for precise neural manipulation of individual cells or brain areas with high spatial precision in animal models. However, in humans, optogenetics-based methods are not viable because they require genetic manipulation, injections are inherently invasive and, whilst deep brain stimulation (Perlmutter and Mink 2006) has been effective in a clinical setting for the treatment of diseases such as Parkinson's disease, it is not viable for probing neural function in healthy volunteers.

The development of non-invasive brain stimulation (NIBS) methods (Polanía et al. 2018) has provided neuroscientists with a tool for modulating neural activity in healthy humans in order to further investigate brain function. The two main established modalities are transcranial electric stimulation (tES) (Nitsche et al. 2008) and transcranial magnetic stimulation (TMS) (Walsh and Cowey 2000). tES consists of placing electrodes on the scalp to deliver weak currents through the brain between the two electrodes. Several variations of this method exist using either direct currents (Nitsche et al. 2008), alternating currents (Herrmann et al. 2013) or random noise (Terney et al. 2008) as the stimulatory input. However, all these approaches result in a highly diffuse electric field that cannot be localized to a specific brain target; reducing the size of one of the electrodes can increase the focality but still results in about 10 cm^2^ of the brain surface area within 50% of the maximum power (Faria et al. 2012). The alternative method, TMS, uses an extracorporeal magnetic coil to produce electric currents inside the brain *via* electromagnetic induction. Again, traditional ring and figure-8 coil designs suffer from diffuse fields (volumes on the order of many cm^3^) that decay exponentially in amplitude from the brain surface with depth, limiting their scope to the cortical surface. At a depth of 1.5 cm, the lateral spread of the magnetic field is over 10 cm^2^ (Deng et al. 2013). Despite these spatial limitations, the functional resolution of TMS is thought to be somewhat higher, as demonstrated by specific motor movements following stimulation of different parts of the motor cortex. More recent coil configurations, for example the H-coil (Zangen et al. 2005), also provide potential for stimulating deeper brain targets. Finally, whilst TMS is a safe method, some TMS stimulation protocols have been associated with discomfort in patients (Rossi et al. 2009).

The most promising electrical modality for stimulating neurons at depth without activating tissue at the brain surface is temporal interference, which uses multiple high-frequency electric fields that do not cause neural activation except where they overlap, the subsequent interference thereby delivering a lower frequency stimulus in the required firing rate range to stimulate neurons (Grossman et al. 2017). To date, this technique has only been demonstrated in mice but should be scalable for use in humans.

The methods described thus far have focused on inducing neural activity through the use of applied electric fields in keeping with the Hogkin-Huxley model of action potential (AP) triggering through electrochemical coupling (Hodgkin and Huxley 1952). However, mechanical forces within the body, and specifically the brain, also play a major role in cell functions, including proliferation, signaling and differentiation (Mueller and Tyler, 2014, Tyler, 2012, Wang and Thampatty, 2006).

Focused ultrasound (FUS) is a way of non-invasively delivering mechanical forces to cells deep within the body in the form of an acoustic pressure wave, which can result in numerous bioeffects, both thermal and mechanical, depending on the specific pulsing regime (ter Haar 2010). The acoustic waves can be focused to a particular location with a spatial resolution on the order of the wavelength of the driving frequency (approximately 3 mm at 0.5 MHz). As the focusing is achieved through constructive interference of the incident waves, a focal spot can be formed at depth within the tissue without affecting cells along the propagation path closer to the transducer.

Therapeutic delivery of ultrasound (US) to the brain was first conducted in the 1950s in order to thermally ablate a distinct volume of tissue resulting in the formation of a lesion (Fry et al. 1955). This therapy, known as high-intensity focused ultrasound or HIFU, permanently destroys a region of tissue and has applications for treating brain cancers (Martin et al. 2014) and other neurologic disorders, such as tremors, whereby ablation of a specific brain area can lead to significant symptom improvements (Lipsman et al., 2014, Wang et al., 2015). Additionally, shorter FUS pulses in combination with intravenously injected US contrast agents (UCAs) can be used to open the blood–brain barrier (BBB) *via* mechanical mechanisms (Hynynen et al., 2005, McDannold et al., 2012, Sheikov et al., 2008) and locally deliver therapeutic agents ranging from small molecule drugs (Treat et al. 2007) to viral vectors (Alonso et al. 2013). HIFU could therefore be utilized for delivering drugs in order to achieve pharmacologic neuromodulation of specific brain targets (Airan et al. 2017).

However, US alone at lower intensities can result in direct neuromodulation of neurons (Khraiche et al., 2008, Tufail et al., 2010, Tyler et al., 2008) without the addition of any other therapeutic agents. Consequently, FUS has a huge potential to become a NIBS method, providing increased spatial selectivity over existing electrically based NIBS protocols coupled with the ability to target areas of the brain at any depth.

The purpose of this review is to summarize work over the past several decades demonstrating the effects of US on neural tissue in both the central and peripheral nervous systems. Most studies cited here were published in journals that currently require authors to have had clinical and/or animal trials approved by the appropriate institutional review board; for those that were not published in mainstream journals, the references were checked to ensure there was a statement to that effect. In addition, we discuss what we believe are the main barriers at present to the uptake of FUS as a viable neuromodulatory tool, namely: an understanding of the mechanistic underpinning of the transduction of the acoustic wave into neural activity modulation; the safety of the technique from both a thermal and mechanical viewpoint; the delivery of US focused to a given brain or nerve target; and treatment monitoring to ensure successful targeting, as well as to record the induced neurologic effects.

## Ultrasound Exposure

### Parameters

**Fig. 1:**
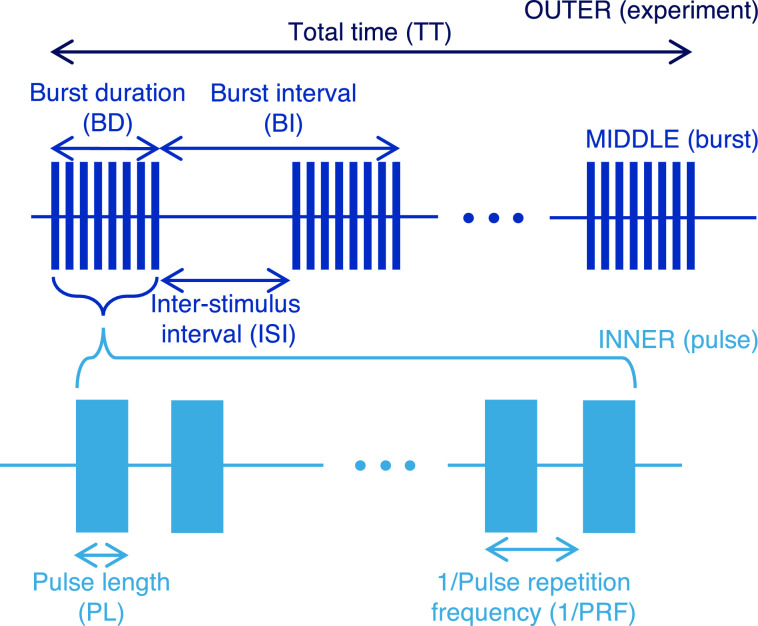
Schematic of ultrasound sequences and associated parameters typically utilized for ultrasonic neuromodulation.

Careful description of the US parameters is key to defining the sequences utilized for inducing ultrasonic neuromodulation. Typically, the sequences are defined over multiple time scales with up to three layers, as shown in Figure 1. At the shortest time scales, or inner layer, are the individual pulses of US. The pulses have an associated pulse repetition frequency (PRF) and are repeated at this frequency for a length of time defined by the burst duration (BD), comprising the middle layer. The burst duty cycle (BDC), or duty cycle over a BD, is therefore the pulse length (PL) multiplied by the PRF. Each burst comprises one distinct trial, and these are delivered at a burst repetition frequency (BRF). The time between each burst is defined as the inter-stimulus interval (ISI). The BRF is thus the inverse of the sum of the BD and ISI. The final, outer layer refers to the total time (TT) of the experiment and has an associated total duty cycle (TDC) that refers to the duty cycle over the whole experiment, accounting for the ISI between bursts.

The variety of time scales present in the problem means that intensity values can also be reported over multiple time scales. Here, the spatial-peak, pulse-averaged intensity (I_SPPA_) will be defined as the average intensity of an individual pulse. The spatial-peak, burst-averaged intensity (I_SPBA_) refers to the intensity averaged over one BD, and the spatial-peak, temporal-averaged intensity (I_SPTA_) is the intensity averaged over the total experimental time, including the ISI. For sequences that do not employ bursts of short US pulses, but rather use longer, continuous wave US pulses, the burst parameters (BD and burst interval [BI]) are not relevant to defining the pulsing sequence and the PL, ISI and TT are sufficient to characterize the sequences. All of the parameters with associated abbreviations and units are shown in Table 1.

**Table 1:** Definitions of ultrasound parameters

Parameter	Abbreviation	Unit
Frequency	f	MHz
Pressure (peak instantaneous)	p	MPa
Intensity: spatial-peak, pulse-averaged	I_SPPA_	W/cm^2^
Intensity: spatial-peak, burst-averaged	I_SPBA_	W/cm^2^
Intensity: spatial-peak, temporal-averaged	I_SPTA_	mW/cm^2^
Pulse length	PL	ms
Pulse repetition frequency	PRF	Hz
Burst duration	BD	ms
Burst repetition frequency	BRF	Hz
Burst duty cycle	BDC	%
Burst interval	BI	s
Inter-stimulus interval	ISI	s
Total duty cycle	TDC	%
Number of pulses/bursts/trials	N	–
Total time	TT	s
Mechanical index	MI	–
Thermal index	TI	–

### Induced effects

US can result in the deposition of both mechanical and thermal energy in the medium that it passes through. Of particular importance to neuromodulation applications are the mechanical effects of acoustic radiation force (ARF) and cavitation. These are both well studied topics (Dalecki 2004). Here, we give a brief synopsis and highlight the key parameters. The ARF occurs because of attenuation in the tissue removing momentum from the wave, which results in a net force on the tissue (Palmeri and Nightingale 2011). For a time-harmonic, progressive, plane wave, the force per unit volume of tissue is given by (Nyborg 1965):

(1) $$F_{ARF}=\frac{2\alpha I}{c_{0}}≃\frac{\alpha_{0}}{\rho_{0}c_{0}^{2}}fp^{2}$$

where *α* is the attenuation, *I* is the local intensity of the acoustic field, *c*_0_ is the speed of sound and *ρ*_0_ is the density. Attenuation in biological tissues follows a power law dependence of the form *α* = *α*⋅*f*^n^, where the exponent *n* varies between 1 and 1.5. Here, we have assumed a linear relation (*n* = 1) in order to estimate the ARF, and hence, in the second form of eqn (1), the ARF can be seen to vary linearly with frequency and with the square of the pressure amplitude, *p*.

Acoustic cavitation is the generation of voids or bubbles within the tissue due to the tensile phase of the acoustic wave exceeding a threshold (Plesset and Prosperetti 1977). Once formed, the cavity oscillates in response to the acoustic wave, which can result in acoustic emissions, jetting and streaming, which can induce bioeffects (Coussios and Roy 2008). The threshold for acoustic cavitation depends on the peak negative pressure, frequency and duration of the US and is also sensitive to the tissue properties. It will be described more in the safety section.

## Central Nervous System

US is capable of eliciting both excitatory and suppressive effects on central nervous system (CNS) tissue, depending on the nature of the pulsing regime incident on the tissue. US-induced suppression of neural activity was first observed in the 1950s (Fry et al. 1958), where evoked potentials were first reversibly, and then permanently, suppressed as the intensity was increased when sonicating for over 20 s. Shorter pulses have also demonstrated suppressive effects, including the temporary dilation of the cat's pupils (Ballantine et al. 1960) and spreading depolarization waves in rats (Koroleva et al. 1986).

Electrophysiologic tools in combination with hippocampal slices provided the first evidence that US could directly stimulate neurons over a range of temporal scales. Local field potential (LFP) recordings measured both enhancement and suppression of electrically evoked field potentials following exposure times of 2–15 min (Bachtold et al., 1998, Rinaldi et al., 1991); microelectrode array recordings revealed increased firing rates of primary hippocampal neurons following 1 ms (Khraiche et al. 2008) and repeated 2–20 μs (Choi et al., 2013, Kim et al., 2017) US pulses; and whole-cell patch clamp recordings of individual *Cornu Ammonis* 1 (CA1) pyramidal neurons confirmed AP firing in response to five short US pulses of a length of 22.7 μs (Tyler et al. 2008). Comparison of LFPs between US and optogenetic stimulation showed strong similarities for pyramidal neuron stimulation, although the amplitude of the US-induced response was 10–20-fold lower (Moore et al. 2015).

Subcellular responses to US have also been reported: sodium and calcium transients were generated following US stimulation, which were subsequently abolished following application of pharmacologic agents indicating US-triggering of voltage-gated ion channels. These transients were also evident in glial cells producing a larger mean fluorescence change of 1.4 compared with 1.14 for the pyramidal neurons, suggesting glia may have an increased sensitivity to US stimulation. Moreover, US induced vesicle exocytosis and synaptic transmission, which further contributed to network activity (Tyler et al. 2008).

Additionally, these excitatory responses have been reported *in vivo*: US targeted to the motor cortex and hippocampus in anaesthetized mice resulted in increased cortical spiking on LFP recordings, as well as a period of after-discharge activity lasting for up to 3 s and containing gamma (40–100 Hz) and sharp-wave ripple (160–200 Hz) band components (Tufail et al. 2010). The application of tetrodotoxin, a voltage-gated sodium channel blocker, strongly attenuated US-evoked activity, consistent with the results from hippocampal slice experiments (Tyler et al. 2008). The activity was accompanied by tail twitches and electromyography (EMG) spikes with lower temporal-averaged intensities (range: 20.6–162.7 mW/cm^2^) and lower frequencies (range: 0.25–0.5 MHz), resulting in more robust EMG responses but without affecting latency times. Finally, the levels of a number of neurotrophic factors in the hippocampus were also significantly enhanced following pulsed US exposure (Lin et al., 2015, Tufail et al., 2010, Yang et al., 2015). Two of these factors, brain-derived neurotrophic factor (BDNF) and glial cell-line–derived neurotrophic factor, raise intriguing questions of whether US can be used to stimulate hippocampal plasticity with implications for probing learning and memory, as well as offering neuroprotective effects for neurodegenerative conditions, such as Alzheimer's and Parkinson's disease (Liu et al., 2017, Zhao et al., 2017).

As well as modulating the expression of BDNF, the level of extracellular neurotransmitters has been shown to be US-dependent with increased serotonin, dopamine and γ-aminobutyric acid (GABA) levels with respect to controls in anaesthetized rats for up to 2 h following a 20 min US exposure at 175 mW/cm^2^ I_SPTA_ (Min et al., 2011b, Yang et al., 2012). It should be noted that the sampling site for the neurotransmitter levels (frontal lobe) was located away from the sonication site (thalamus). Therefore, these findings highlight the ability to use US not only to transiently alter neuronal activity through increased spiking as has previously been shown (Tufail et al., 2010, Tyler et al., 2008) but also to produce longer lasting effects that affect the global connectivity of the brain, possibly through modulation of the inhibitory GABAergic pathway (Min et al. 2011b).

Numerous studies have focused on stimulating the motor cortex in rodents (Gulick et al., 2017, Han et al., 2018, Kamimura et al., 2016, Kim et al., 2014a, King et al., 2013, King et al., 2014, Li et al., 2016, Mehić et al., 2014, Ye et al., 2015, Yoo et al., 2011b, Younan et al., 2013) with EMG recordings and motor movements, primarily paw and whisker motion, providing quantification of the robustness of a response. This has enabled parametrization studies to be carried out to determine more effective stimulus parameters. One key finding is that the threshold intensity required to produce an EMG spike increases with the carrier frequency of the US at frequencies in the low-megahertz range, as demonstrated by a reduced EMG amplitude as frequency was raised from 0.25 to 0.5 MHz (Tufail et al. 2010); increased success rates (the ratio of contractions to the total number of sonication trials) at lower frequencies for a fixed intensity between 0.25 and 0.6 MHz (King et al. 2013); reduced threshold intensities at 0.35 MHz compared to 0.65 MHz (Kim et al. 2014a); and increasing intensities to provide a given success rate over a much wider frequency band of 0.3 to 2.9 MHz, albeit with a flatter profile in the sub-megahertz range (Ye et al. 2015). This frequency dependence can be accounted for by two different explanations: a cavitation-based mechanism as the cavitation threshold increases with frequency (Church et al. 2015) or as the result of reduced focal spot sizes with increased frequency. Therefore, if the response is non-linear such that the local activity reaches a maximum, the volume of activated tissue may drive the overall motor outcome. A model incorporating these two factors showed good agreement with *in vivo* mouse data consistent with an ARF mechanism, whereby higher frequencies are actually more effective at inducing a local response but activate smaller volumes of tissue, thus requiring higher intensities to modulate behavior (Menz et al. 2017). However, this theory was unsupported by results comparing planar and focused transducers at low frequencies, 0.3–0.6 MHz (Ye et al. 2015).

Other US parameters have also been studied. Increasing the PRF in the range of 100–3000 Hz resulted in greater responses (King et al. 2013) and a BDC of 50%, and BD of 300 ms over a range of PLs, 1–5 ms, yielded the lowest intensity threshold for eliciting a motor response (Kim et al. 2014a).

Moreover, the studies do provide conflicting evidence over a number of points; for example, one group claims pulsed US (Kim et al. 2014a) is the most effective at producing motor responses, whereas another suggests continuous wave US is better (King et al. 2013). The relationship between temporal-averaged US intensity and response amplitude or success rate also varies with the correlation found to be negative (Tufail 2010), flat with all-or-nothing responses (King et al. 2013), or positive (Kamimura et al., 2015, Mehić et al., 2014).

The precise targeting of specific parts of the motor cortex has led to differing muscular outputs. The average latency times significantly reduced for both neck and tail EMGs when moving the US focus from rostral to caudal brain locations, whilst the amplitudes of the EMG signal decreased for the neck but increased for the tail (King et al. 2014), pointing to more complex interactions between the acoustic field and induced brain network activity. Higher frequencies may be beneficial in this regard, as they can pinpoint smaller brain targets offering increased anatomic specificity; 5 MHz US was shown to be effective at inducing EMG responses, despite the observed trend that higher frequencies require higher intensities to induce activity, and resulted in much shorter latency times versus 1 MHz (45 ms and 208 ms, respectively) (Li et al. 2016).

Additionally, much higher frequency US (43 MHz) has been demonstrated to mediate modulatory effects. In isolated salamander retinas, US evoked strong responses in ganglion cells that were independent of the PL and PRF above 15 Hz (Menz et al. 2013). Activity was only correlated with temporal-averaged intensity and saturated above 10–30 W/cm^2^. Following the blockade of synaptic transmission, the induced effects were no longer evident, indicating that US did not directly activate ganglion cells and thus requires synaptic transmission. At 43 MHz, cavitation activity is unlikely and an ARF-based mechanism provides a better description of the data, particularly as on and off responses were recorded from US pulses. Conversely, at 0.2 MHz, motor movements due to US exposure were associated with a 3 s refractory period, providing evidence that a recovery time of an US-specific mechanism was required, consistent with a cavitation mechanism (Gulick et al. 2017). Consequently, it might be that at different frequencies, different mechanisms exist for coupling acoustic energy into neural activity.

Similarly to TMS, the functional specificity of FUS-induced neuromodulation may be somewhat smaller than the intensity or pressure full-width half-maximum focal volumes. Glucose uptake, as measured by positron emission tomography imaging, was used to assess induced responses, and the activated region was reported to correspond to the full-width 90%-maximum of the intensity field (Kim et al. 2014b).

In general, whilst these studies have uncovered certain trends, the results are highly variable. The explanation is likely to be multi-faceted with differences in experimental setup, anatomic variations and complex interactions between either the stimulation, or inhibition, of competing inhibitory and stimulatory networks, all contributing to the high variance in outcomes. Another crucial parameter that has been shown to significantly alter experimental outcomes is the depth of anesthesia the animals are under during the stimulation. In particular, many US-induced responses are only evident under a light state of anesthesia (King et al., 2013, Younan et al., 2013). A more in-depth study looking at ketamine revealed that US-induced motor responses were abolished for more than 20 min following its injection. This was attributed to ketamine blocking US-triggered calcium transients, as shown by fluorescence imaging of cortical cell cultures (Han et al. 2018). It has also been reported that US reduced anesthesia times following administration of a single anesthetic dose by 20 min, as measured by pinch response and voluntary movement (Yoo et al. 2011b) and even, remarkably, could awaken an individual from a coma (Monti et al. 2016).

Although the motor cortex has been the subject of the majority of studies in small animal models, the visual system has been studied in small animals as well as in larger animals and humans, allowing electrophysiologic measurements (electroencephalography [EEG]) and imaging techniques (functional magnetic resonance imaging [fMRI]) to be combined with behavioral and cognitive assessments. Visual-evoked potentials (VEPs) were suppressed in a rabbit model following pulsed sonication at 0.69 MHz. The effect lasted for 10 min, and the suppressive outcome was confirmed by blood oxygenation level–dependent (BOLD) fMRI (Yoo et al. 2011a). Similar responses were revealed in rats along with VEP enhancement through adjustment of the TDC or intensity of the US sequence (Kim et al. 2015). Below 1% TDC, the VEP was not modulated, indicating a threshold for observing a response, which is consistent with findings from the motor cortex (King et al. 2013). At 5%, the magnitude of the P1 component minus the N1 component was reduced by approximately 13%, whereas increasing the duty cycle to 8.3% or the I_SPPA_ from 1 to 5 W/cm^2^ led to an increase of P1-N1 close to 10%.

Comparable electrophysiologic measurements were recorded in both pigs and sheep. In sheep, repeated stimulation of both primary visual and sensorimotor areas was explored, leading to EMG and EEG responses, respectively, (Lee et al. 2016c), and in pigs, thalamic targeting also led to reversible suppression of somatosensory-evoked potentials (SEPs) (Dallapiazza et al. 2018).

In awake monkeys, US was targeted to the frontal eye field (FEF) during an antisaccade task (Deffieux et al. 2013). Ipsilateral mean latencies were significantly slowed following FUS exposure, although the same results were not observed in prosaccade tasks. These results demonstrated for the first time the ability to use FUS as a neurostimulation tool to modulate high-level cognitive behavior (Deffieux et al. 2013). A second paper by the same group measured multiple single neuron recordings in a connected brain region, paving the way for future studies investigating network connectivity changes (Wattiez et al. 2017). They recorded significant modulation of approximately 40% of neurons in this connected region, closely matching the reported value in hippocampal slices that 30% of neurons were stimulated, albeit in this instance, the stimulation and recording sites were coincident (Tyler et al. 2008). Behavioral modulation following right-side FEF sonication during a visual task was also observed shifting animals’ choices to the leftward target and vice versa, left-side FEF stimulation shifted choices to the rightward target (Kubanek et al. 2017).

Based on the evidence from animal models (Tyler et al., 2008, Yoo et al., 2011a), it has been suggested that FUS would be a safe method to be used in humans. The first US stimulation study for neuromodulation of the human brain was conducted with a diagnostic imaging probe operating at 8 MHz placed over the posterior frontal cortex for 15 s (Hameroff et al. 2013) and consequently led to significant improvements in mood, but not pain, scores in chronic pain volunteers. At this frequency, very little of incident acoustic energy is likely to penetrate the skull, and so subsequent studies have all focused on sub-megahertz frequencies utilizing US sequences very similar to those found to be the most effective in small animal parametrization studies.

Following successful median nerve stimulation *via* US, which activated somatosensory pathways within the brain (Legon et al. 2012) (see Peripheral Nervous System section), the next step was to try and modulate the induced SEPs with US targeted to the primary somatosensory cortex (S1) in healthy volunteers (Legon et al. 2014). Attenuation of the SEP was reported along with specific modulation of alpha (7–12 Hz), beta (13–20 Hz) and gamma (30–55 Hz) frequency bands at an US frequency of 0.5 MHz, 500 ms BD at a 36% BDC and an I_SPPA_ of 5.9 W/cm^2^. Moreover, improvements in two-point touch and frequency discrimination tasks followed FUS sonication. Further analysis of the EEG data revealed modulation of both intrinsic and evoked EEG dynamics (Mueller et al. 2014). Overall, these findings demonstrated the use of FUS to non-invasively modulate cortical processes in humans.

Specifically targeting the hand S1, secondary somatosensory cortex, or both areas simultaneously with FUS was able to induce peripheral sensations in volunteers (Lee et al., 2015, Lee et al., 2016a). The reported perceptions varied from tingling and numbness to itching and coolness, as well as ranging in their location from fingertips all the way up to the axilla. The distribution in location of the induced peripheral sensations points to the misalignment of the US focus and the target location and highlights a key problem in scaling up from smaller animals to humans. This difficulty in ensuring correct targeting is further discussed in the Delivery section. A similar issue was also seen in a study delivering US to the primary visual cortex, where retrospective simulations revealed misalignment problems in some participants. However, in the volunteers where the US was correctly focused, 300 ms US bursts at a 50% BDC, driving frequency of 270 kHz and I_SPPA_ ranging between 1.2–6.6 W/cm^2^, induced phosphene perception, which was associated with EEG modulation (Lee et al. 2016b). Concurrent fMRI maps confirmed activation of the target site as well as showing activity in connected visual and higher order cognitive pathways. A similar pattern of activation has been observed following phosphene induction *via* TMS (Lee et al. 2016b).

Combined magnetic and US stimulation has been used to examine US modulatory effects in the human motor cortex (M1) (Legon et al. 2018b). US was paired with a number of established TMS protocols, and a burst of 500 ms at 500 kHz was delivered 100 ms before the TMS pulse, which attenuated single-pulse motor evoked potentials (MEPs). In paired pulse protocols, two TMS pulses are delivered at a specified time interval, which determines if the MEP is inhibited or promoted. For short intervals, the MEP is inhibited (short interval intra-cortical inhibition [SICI]), whereas for longer intervals, it is facilitated (intra-cortical facilitation [ICF]) (Ziemann et al. 1996). US attenuated ICF but had no effect on SICI. The cortical silent period, an interruption of voluntary muscle contraction following M1 stimulation (Wilson et al. 1993), was not affected by US stimulation, but US did reduce reaction times in a stimulus response task (Legon et al. 2018b).

The simultaneous acquisition of fMRI data from both 3 T and 7 T MRI scanners in conjunction with FUS stimulation to cortical and sub-cortical regions has been explored (Ai et al. 2016). Whilst image artifacts from the FUS source cannot be completely eliminated, they can be reduced to an acceptable level in order to detect cortical activity close to the transducer. Moreover, targeting of deeper, thalamic regions also suppressed SEP components along with time-locked gamma band (approxiately 80 Hz) inhibition and a reduction in performance for a two-point discrimination task (Legon et al. 2018a).

Neuromodulatory effects have also been observed following the intravenous injection of UCAs and US sonication for BBB opening (Chu et al., 2015, Downs et al., 2015). In rats, 400 kHz US at a mechanical index (MI) of 0.55 (0.35 MPa) with injected microbubbles produced transient SEP amplitude modulation (less than 1 h) without affecting latency times or inducing damage. However, increasing the MI to 0.8 (0.51 MPa) resulted in red blood cell extravasation and was associated with prolonged SEP amplitude and latency suppression. However, without microbubble injection, 0.8-MI US did not induce BBB opening. (Chu et al. 2015). Furthermore, in monkeys, BBB opening was associated with a significant error reduction during a visuomotor task (Downs et al. 2015). Although this protocol, where agents are injected, is not viable for healthy volunteers, these results suggest a possible combined beneficial effect of BBB opening not only to deliver a pharmacologic agent but also to produce a direct behavioral change.

Alternative exogenous agents have also been developed to sensitize neurons to the applied US field: piezoelectric nanoparticles couple acoustic energy into an electric field, generating calcium transients (Marino et al. 2015) and network activity (Rojas et al. 2018) at pressures as low as 1–2 kPa.

Finally, analogous to optogenetics (Fenno et al. 2011), the term sonogenetics has been coined and investigated through genetic manipulation of *Caenorhabditis elegans* with (Ibsen et al. 2015) and without microbubbles (Kubanek et al. 2018). Expression or mis-expression of specific ion channel subunits found in touch-sensitive neurons led to US-induced behavior and therefore offer the potential to selectively and non-invasively stimulate only genetically altered neurons without requiring surgical intervention to deliver light as in optogenetic approaches.

## Peripheral Nervous System

Ultrasonic neuromodulation applied to the peripheral nervous system (PNS) has been conducted in parallel to the work in the CNS. At a similar time to the first studies noticing reversible effects in the brain, it was discovered that US sonication of the peripheral nerves could first increase spiking activity and then depress spontaneous activity in an initially reversible, and then permanent, manner (Fry et al., 1950, Lele, 1963, Young and Henneman, 1961). At the same time, conduction velocities increased with the applied ultrasonic dose. Both of these results were replicated by heat application (Lele 1963), indicating a thermal mechanism. Differential blocking of mammalian nerves has also been observed with the smallest, C fibers, being the most responsive and the largest, A-*α*, being the least sensitive (Legon et al., 2012, Lele, 1963, Young and Henneman, 1961). As the C fibers carry pain signals from receptors, this opens the obvious question as to whether US can be used to suppress pain.

A number of more recent studies confirmed these findings, showing that evoked potential amplitudes could be initially increased by up to 9% before decreasing at higher intensities (Colucci et al., 2009, Foley et al., 2008, Tsui et al., 2005). As previously, these long pulse (5 s to 5 min) sonications are primarily attributed to thermal effects. Short pulses (0.5 ms) of US have also been shown to modulate electrically induced compound action potentials (CAPs) with either early enhancement or suppression, depending on the latency time between the applied US pulse and electrically induced CAP (Mihran et al. 1990). These pulses have an estimated temperature rise of 0.025°C, and so a thermal mechanism is unlikely to be driving the observed responses, suggesting mechanical effects may be involved. Reductions in conduction velocities (Juan et al., 2014, Wahab et al., 2012;) also point to a non-thermal mechanism, as the conduction velocity is expected to increase with temperature (Lele 1963).

Both ARF and cavitational-based mechanisms have been proposed to account for US-mediated PNS modulation: the cumulative radiation force was shown to negatively correlate with reductions in both conduction velocities and AP amplitudes following electrical stimulation *via* an electrode (Wahab et al. 2012), whereas in *ex vivo* crab axons, *de novo* APs could only be excited when cavitation signatures were simultaneously acquired on passive cavitation detectors (Wright et al., 2015, Wright et al., 2017). It should be noted that the minimum pressure required to induce direct AP generation was 1.8 MPa at 0.67 MHz, on the order of a magnitude higher than the lowest pressures required to modulate electrically induced APs (0.1 MPa) (Wahab et al. 2012).

An *in vivo* study in mice also required higher pressures (a minimum of 3.2 MPa) to induce EMG activity and visible muscle responses following sonication of the sciatic nerve at 3.57 MHz. A break period of 20–30 s improved the success rate of subsequent stimulation to 92% (Downs et al. 2018). This regeneration phase is similar to that reported in the CNS targeting US to motor cortex where a 3 s refractory period was described (Gulick et al. 2017). Moreover, shock waves of 50 MPa in amplitude are capable of directly generating CAPs with a similar shape to electrically induced CAPs, but lower amplitudes (Schelling et al. 1994). Movement of the shock wave focus away from the nerve abolished CAP generation until air bubbles were injected. Overall, these results lend support to a cavitational-based mechanism for generating *de novo* APs in peripheral nerves.

However, at 350 kHz, pressures of only 0.53 MPa induced eyeball movements in rats following sonication of the abducens nerve (Kim et al. 2012). The extent of the movement grew after each 200 ms burst for 10 repeated bursts at a BI of 1 s. Although at a higher frequency of 650 kHz, movements could not be induced.

In injured animals models, neuropathic tissue has been shown to be more sensitive to US than healthy tissue (Tych et al. 2013), as well as to improve regeneration and recovery following crush injury linked with increased BDNF levels (Ni et al. 2016). US has also shown benefits in the treatment of other diseases. For example, in bladder dysfunction, US inhibited rhythmic bladder contractions with longer latencies and refractory periods compared with electrical stimulation (Casella et al. 2017).

Gavrilov et al. made a series of pioneering contributions to the field. Initial experiments in the Pacinian corpuscle, a mammalian mechanoreceptor, showed the induction of APs following US exposure at 0.48 MHz between 0.4 and 2.5 W/cm^2^, with increasing potential amplitudes as the intensity was increased (Gavrilov et al. 1977b). Translating these results into humans, a range of tactile sensations were elicited following short US pulses (1–100 ms) targeted to the hand or forearm (Gavrilov et al. 1977a). As the PL was further increased, sensations were present at the start and end of the waveform as well as estimated displacements matching mechanical displacements required to stimulate receptors, indicating the ARF might be the mechanistic driving force behind the sensations (Gavrilov 1984). With increasing intensity, the sensations also changed in nature from tactile to temperature and finally to pain perception. Before US intensities reached the level to cause the onset of pain, cavitation signals were also detected. In auditory nerves, it was shown that sonication led to evoked potentials in the brain at intensities as low as 0.01 W/cm^2^ and with a similar form to those induced by sound stimuli (Gavrilov et al. 1977b). Consequently, it was postulated that US may have applications to both diagnosing neurologic diseases based on US tactile sensation response as well as the encoding of auditory information for the deaf.

A hypothesis that variability in the intensity required to elicit tactile sensations in different tissues and individuals was related to the density of mechanoreceptors, made initially by Gavrilov et al. (1984), was supported by a subsequent study in humans (Dickey et al. 2012). The sensitivity of individuals showed a sigmoidal relationship with respect to intensity, with an average intensity of 106.4 W/cm^2^ to reach a 90% threshold response rate at 1.1 MHz.

Modifying the sequence parameters could also change the induced sensations (Lee et al., 2014, Legon et al., 2012) and their associated brain activity as quantified by EEG and BOLD fMRI maps showing activation of different cortical and sub-cortical regions, depending on the pulse incident on the fingertip (Legon et al. 2012). Although thermal sensations were maximal over a band of intensities (I_SPTA_ = 10–30 W/cm^2^), vibrotactile and nociception responses continued to increase for the range of intensities tested, up to 100 W/cm^2^ (Lee et al. 2014).

## Mechanisms

Although historically nerve impulses have often been considered as electrical signals, where depolarization of the membrane beyond a threshold potential leads to excitation, it is now recognized that nerve impulses involve a combination of electrical, mechanical, chemical and conformational changes in the excited cells (Abbott and Howarth, 1973, Bose, 1902, Bulychev et al., 2004, Cohen and Salzberg, 1978, Luzzati et al., 1999, Tasaki, 1982, Tasaki, 1995, Terakawa, 1985). Excitation and inhibition of nerve impulses has been reported in response to electrical (Hodgkin and Huxley 1952), chemical (Fillafer and Schneider, 2016, Newman and Zahs, 1998, Tasaki et al., 1962), mechanical (Bose, 1902, Guharay and Sachs, 1984, Julian and Goldman, 1962, Newman and Zahs, 1998, Spyropoulos, 1957) and thermal (Chapman, 1967, Franz and Iggo, 1968, Guttman, 1966, Inoue et al., 1973, Shapiro et al., 2012) stimuli. The presence of a mechanical pathway provides a physical basis for ultrasonic neuromodulation, and here we will review four potential mechanisms by which US could result in subsequent triggering of APs: (i) the generation of capacitive currents due to membrane displacements, (ii) the activation of mechanosensitive channels, (iii) the opening of pores in the lipid bilayer, so-called sonoporation and (iv) coupling into membrane waves along the axon. There is overlap in the physical basis by which these mechanisms occur, and so it may be that it is a combination of these mechanisms, and potentially others, that provides a means for US to effect neurostimulation.

US waves carry energy and can do work on and exchange heat with the medium they propagate through. The principal interface by which US affects neurons (and indeed cells in general) is through interaction with cell membranes, the biophysics of which is a rich and mature field (Lipowsky, 1995, Sackmann, 1995). From a physical chemistry perspective, there is comprehensive literature on the excitability of a nerve that describes the phenomenon as a phase transition associated with a change in the conformational state of the plasma membrane (Abbott and Howarth, 1973, Inoue et al., 1973, Kobatake et al., 1971, Luzzati et al., 1999, Margineanu and Schoffeniels, 1977, Tasaki, 1959, Ueda et al., 1974). The phase transitions that can occur in biological membranes are very diverse (Georgescauld et al., 1979, Hazel et al., 1998, Koynova and Caffrey, 1998) and have time-scales from ns to ms (Holzwarth 1989). Within that range of time scales, US has been shown to effect changes in the conformational state of single and multiple component lipid vesicles and proteins (Halstenberg et al., 1998, Holzwarth, 1989, Kessler and Dunn, 1969, O'Brien Jr and Dunn, 1972, Tatat and Dunn, 1992). The mechanisms described here are related to processes by which US produces conformational changes, which will then result in nerve excitation.

### Membrane capacitance: flexoelectricity and conformational changes

The original Hodgkin and Huxley model (1952) of the propagation of nerve impulses in a neuron modelled the membrane as a fixed capacitance. Subsequent studies have shown that changes in the membrane properties, such as thickness (Heimburg 2012), curvature (Petrov 2002) and the conformational state of the lipids in the membrane (Antonov et al., 1985, Taylor et al., 2017), result in changes in capacitance, which can result in excitation of nerve impulses (Heimburg, 2012, Luan et al., 2014, Plaksin et al., 2014, Plaksin et al., 2017, Shapiro et al., 2012, Zecchi et al., 2017). US has been previously shown to induce capacitive currents in pure lipid membranes (Prieto et al. 2013), which can be explained on the basis of flexoelectric effects or conformational changes. If the perturbations are sufficiently compressive, then the resulting currents would be excitatory, and if dilational, the currents would be inhibitory. An alternative hypothesis is that nucleation and expansion of cavities within, or near, a lipid bilayer could result in capacitive currents (Plaksin et al. 2014), although the exact role of cavitation in ultrasonic nervous stimulation is yet to be fully elucidated.

### Mechanosensitive channels

The activity of ion channels can also be modulated by changes in the conformation state of channel proteins, as well as that of surrounding lipids and other macromolecules (Perozo et al., 2002, Seeger et al., 2010, Sotomayor et al., 2000). Numerous different ion channels are mechanosensitive (Morris 2012) and have been shown to exhibit sensitivity to US of varying degrees (Brohawn, 2015, Morris, 2011, Mueller and Tyler, 2014). Specific channels that appear to respond to US stimulation include: two pore domain K^+^ channels (Kubanek et al. 2016), Na_v_1.5 channels (Kubanek et al. 2016), voltage-gated Na^+^ and Ca^2+^ channels (Tyler et al. 2008), transient receptor potential channels (Ibsen et al., 2015, Li et al., 2018) and Piezo1 channels (Pan et al., 2018, Prieto et al., 2018). In the case of Ca^2+^, it is a critical messenger molecule, which is also involved in neuronal function through synaptic activity modulation and through extensive signaling pathways (Brini et al. 2014). Ca^2+^ flows have also been shown to couple to the conformational state of the membrane, where changing either component invariably affects the other (Tasaki 1982). In addition to ion channels, synaptic activity (Borrelli et al., 1981, Tufail et al., 2010, Tyler et al., 2008, Vladimirova et al., 1994) is known to be sensitive to mechanical cues (Siechen et al., 2009, Tasaki, 1995), and glial cells have been shown to respond to US modulation (Kovacs et al., 2017, Newman and Zahs, 1998, Tyler et al., 2008).

### Sonoporation

We define sonoporation as the opening of pores or other transport processes *via* acoustic stimulation that are separate from the ion channels normally employed by the cell membrane. Sonoporation can occur through the creation of physical pores in the bilayer, which would provide a new channel for ion transport, driven by the gradients across the cell membrane. The probability of forming a pore in the membrane has been shown to be directly related to the compressibility and specific heat of the membrane (Antonov et al., 1985, Blicher et al., 2009, Kaufmann et al., 1989, Wunderlich et al., 2009). The specific heat has a local peak at phase transition, and therefore if the US perturbation can nudge the membrane through a transition, then the rate of pore formation will increase (Tatat and Dunn 1992).

Even without the creation of physical pores, the permeability of the membrane can change with its conformation state (Nagle and Scott, 1978, Walter and Gutknecht, 1986, Yang and Kindt, 2015), which affects the solvent environment in the hydrophobic core (Koynova and Caffrey 1998). Therefore, if US alters the conformational state of the membrane, the permeability will adjust, resulting in changes to the gradient-driven ion currents (Tatat and Dunn 1992).

We note that in the presence of a microbubble, neurostimulation by US seems to be enhanced, potentially by pore formation. For example, intracellular Ca^2+^ waves have been observed following microbubble collapse (Li et al. 2018), with a fast wave occurring when pores in the membrane were created by the microbubble collapse but a slower wave when it was not. The Piezo1 Ca^2+^ channel has also been observed to be activated by 2 MHz US, but only in the presence of microbubbles (Pan et al. 2018). However, the focus of most neurostimulation studies has been to avoid the use of microbubbles as they increase the risk for injury.

### Membrane waves

Recent experimental and theoretical research has established that nerve impulses are associated with elastic interface waves that propagate along the wall of the axon or the plasma membrane (El Hady and Machta, 2015, Kim et al., 2007); the propagation of the mechanical disturbance is coupled to ionic currents and chemical potentials (Fichtl et al. 2016). Experiments in lipid membranes have shown that when the membrane is close to a phase transition, the interface waves behave in a manner that is strikingly similar to nerve impulses, including a threshold for excitation (Shrivastava et al., 2015, Shrivastava and Schneider, 2014), velocities similar to nerve conduction in unmyelinated neurons (Kappler et al. 2017) and annihilation upon collision (Shrivastava et al. 2018). Interface waves can be stimulated mechanically and result in coupled electrical potentials in neurons (El Hady and Machta 2015), which has led to the suggestion of interface waves as the physical basis for nerve impulses and biological signaling (Andersen et al., 2009, Fichtl et al., 2016, Griesbauer et al., 2012, Heimburg and Jackson, 2005, Shrivastava and Schneider, 2014), although these ideas are not yet mainstream. Therefore, if an US wave couples into an interface wave in the axon, that can lead to the corresponding chemical (Fichtl et al. 2016) and electrical processes that result in a nerve impulse (Andersen et al., 2009, Wunderlich et al., 2009). Alternatively, the acoustic perturbation could move the interface far enough away from transition to suppress a nerve impulse.

### Thermal effects

Alongside the mechanically induced effects, absorption of the US wave leads to temperature rises, which may also result in thermal neuromodulation dependent on the incident waveform. A similar thermal absorption mechanism is used to achieve infrared neural stimulation (Chernov and Roe 2014). Temperature changes on the order of a few degrees can affect neural activity, altering the amplitude and duration of APs, excitation thresholds, spiking rates and afterhyperpolarization kinetics, (Chapman, 1967, Guttman, 1966, Lee et al., 2005, Thompson et al., 1985). Certain ion channels are also known to exhibit thermosensitivity (Cesare et al. 1999).

### Concluding remarks

Although nerve impulses are often thought of as electrical signals, in reality they involve mechanical, thermal, chemical and conformational changes in the plasma membrane as well. Here, we have described how acoustic perturbations have the potential to couple to these various aspects of cellular excitability and alter the conformational or thermodynamic state of the plasma membranes of cells, which could result in sufficient depolarization to trigger a nerve impulse or to suppress depolarization and inhibit nerve firing. The excitatory or inhibitory actions can be through changes in membrane capacitance, changes to ion channels, the opening of pores and coupling to interfacial elastic waves. In addition to conformational changes in the cell membrane, flexoelectricity and state change due to cavitation also have the potential to contribute to neurostimulation.

A key aspect to the conformational changes is that the cell membrane is sitting close to a thermodynamic phase transition, which means even small perturbations can cause significant structural changes that lead to nerve firing (Tasaki 1982). In the context of human CNS modulation, given the typical parameters employed, the most likely mechanism by which modulation occurs is by ARF (Menz et al., 2017, Mihran et al., 1990, Prieto et al., 2018) as it is sufficient to deform tissue and has time scales better matched to the underlying conformational changes (Holzwarth, 1989, Tatat and Dunn, 1992). On the other hand, PNS stimulation associated with higher intensities may require other bioeffects to induce a response, such as cavitation. For sufficient radiation force, the conformational change will directly polarize the membrane *via* flexoelectric (Petrov 2002) or mechanocapacitive (Zecchi et al. 2017) coupling, resulting in immediate excitation without delay. At lower amplitudes, the radiation force will depolarize the membrane by slowly depleting the ion gradients (rheobasic current), and the duration will need to be sufficiently long for polarization to cross the threshold required to produce a nerve impulse.

We have focused on the four mechanisms we consider the strongest candidates to affect neurostimulation, although other hypotheses exist, such as the Orchestrated Objective Reduction theory (Hameroff and Penrose 2014), whereby US interacts with microtubular oscillations. Given the complexity of biological systems, it is highly likely that multiple mechanisms play a role in transducing acoustic perturbations into nerve impulses and that the relative contributions may change depending on acoustic parameters (amplitude and time scales) and cell types. The role of cell types, in particular glial cells, demands attention, given significant differences observed in US parameters that result in excitation in CNS versus PNS stimulation. Glial cells fill the space between neuronal elements in the brain and, traditionally, are believed to provide a soft connective tissue that provides structural support (Pogoda and Janmey 2018). However, recent research suggests a more critical role of these cells in the brain for neuronal function (Fields, 2015, Min and Nevian, 2012) as well as functioning as a source and mediator for calcium waves (Chesler, 2003, Newman and Zahs, 1998). Mechanical stimulus is known to be efficient at exciting calcium waves in glial cells, making them a potential target for US stimulation of the brain (Newman and Zahs 1998).

## Safety

**Fig. 2:**
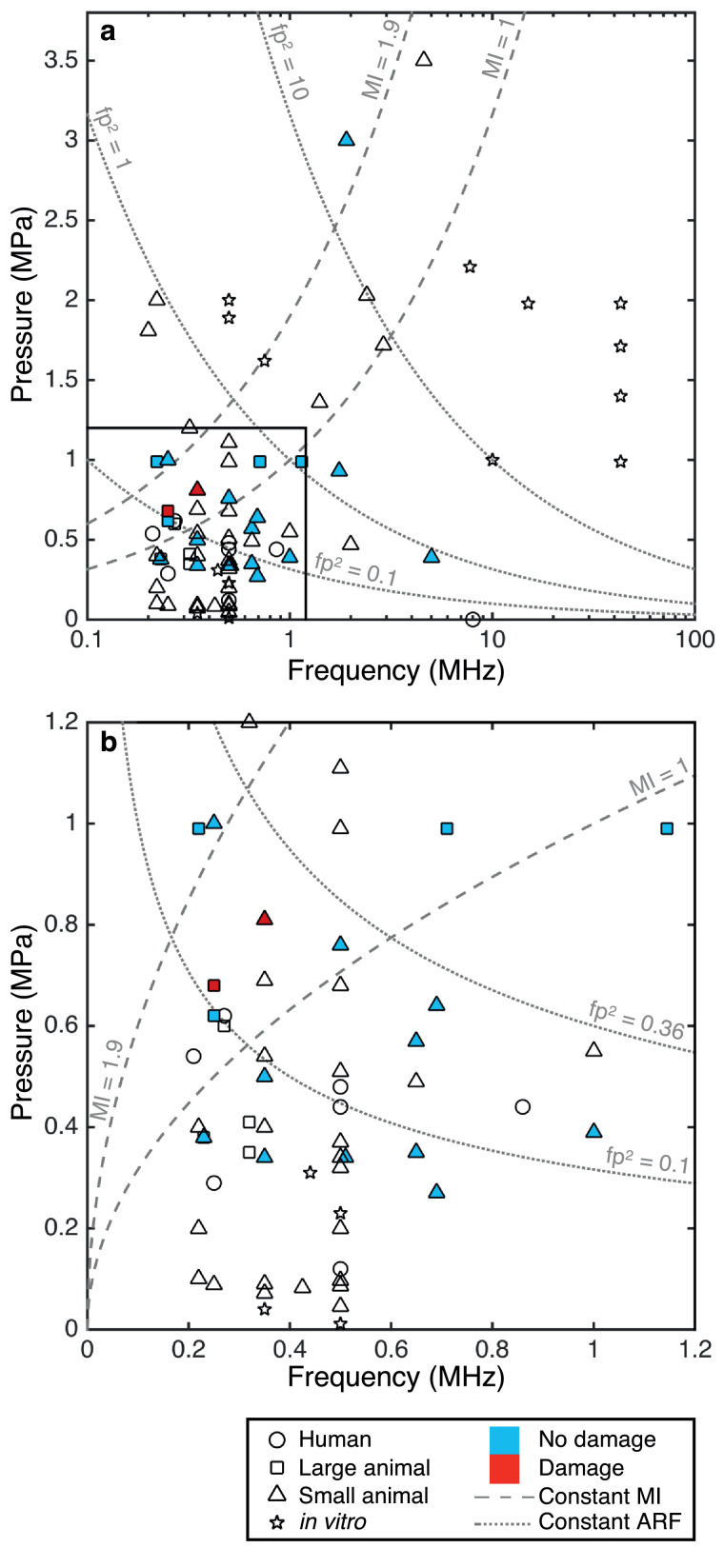
Parameter analysis of central nervous system studies: instantaneous peak pressure (*p*) against driving frequency (*f*). The studies are split into human, large animal, small animal and *in vitro* work. Any studies that conduct histologic analysis and report no damage are filled in blue, and those that report damage are filled in red. Lines of constant mechanical index (MI) and *fp*^2^, a measure of acoustic radiation force (ARF), are also displayed. (a) Full parameter space (log scale). (b) Subset of parameters applicable to transcranial human delivery (linear scale, *p* < 1.2 MPa, *f* < 1.2 MHz). ARF = acoustic radiation force; *f* = driving frequency; *fp*^2^ = measure of acoustic radiation forces; MI = constant mechanical index; *p* = instantaneous peak pressure.

**Fig. 3:**
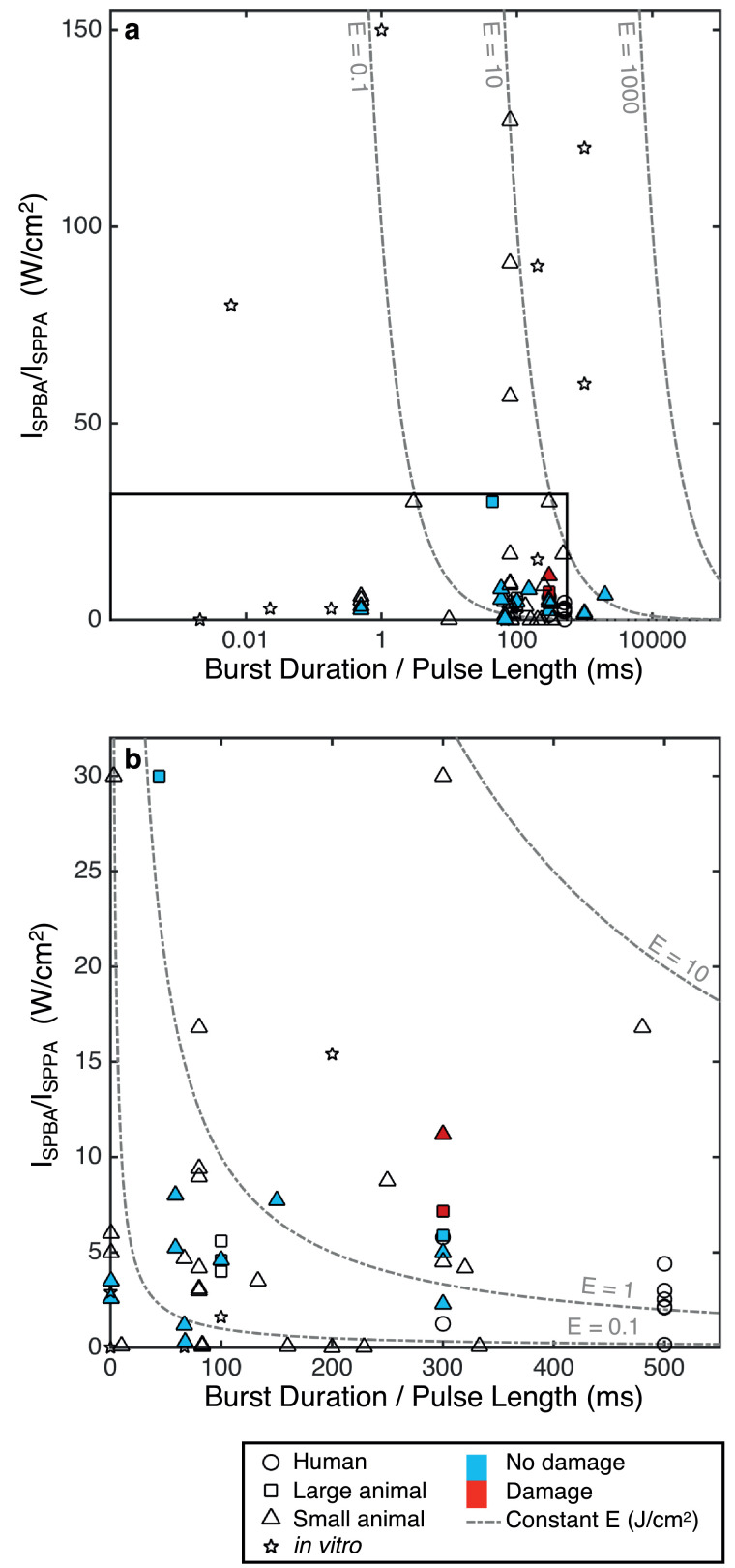
Parameter analysis of central nervous system studies. For burst wave: burst-averaged intensity (I_SPBA_) versus burst duration (BD). For continuous wave: pulse-averaged intensity (I_SPPA_) against pulse length (PL). The studies are split into human, small animal and *in vitro* work. Any studies that conduct histologic analysis and report no damage are filled in blue, and those that report damage are filled in red. Lines of constant energy density (I_SPBA_ × BD or I_SPPA_ × PL) are also displayed. (a) Full parameter space (log scale). (b) Subset of parameters (linear scale, I_SPBA_ / I_SPPA_ < 30 W/cm^2^, BD / PL < 500 ms). BD = burst duration; E = energy density; I_SPBA_ = spatial-peak burst-averaged intensity; I_SPPA_ = spatial-peak pulse-averaged intensity; PL = pulse length.

**Fig. 4:**
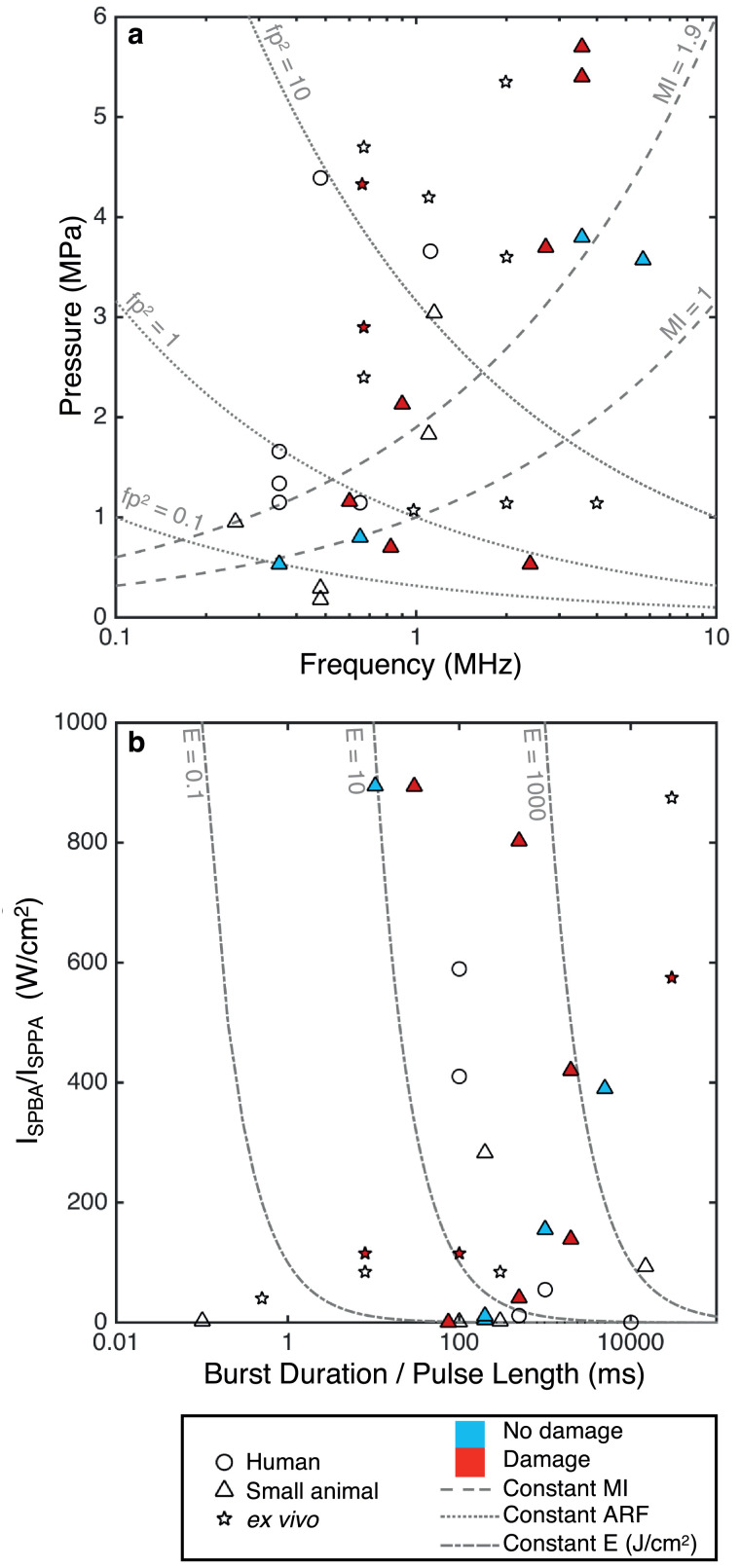
Parameter analysis of peripheral nervous system studies. (a) Instantaneous peak pressure (*p*) against driving frequency (*f*) with lines of constant mechanical index (MI) and *fp*^2^, a measure of acoustic radiation force (ARF) (log scale). (b) For burst wave: burst-averaged intensity (I_SPBA_) versus burst duration (BD). For continuous wave: pulse-averaged intensity (I_SPPA_) against pulse length (PL). Lines of constant energy density (I_SPBA_ × BD or I_SPPA_ × PL) are also displayed (log scale). The studies are split into human, small animal and *ex vivo* work. Any studies that conduct histologic analysis and report no damage are filled in blue, and those that report damage are filled in red. ARF = acoustic radiation force; BD = burst duration; E = energy density; *f* = driving frequency; *fp*^2^ = measure of acoustic radiation forces; I_SPBA_ = spatial-peak burst-averaged intensity; I_SPPA_ = spatial-peak pulse-averaged intensity; MI = mechanical index; *p* = instantaneous peak pressure; PL = pulse length.

**Fig. 5:**
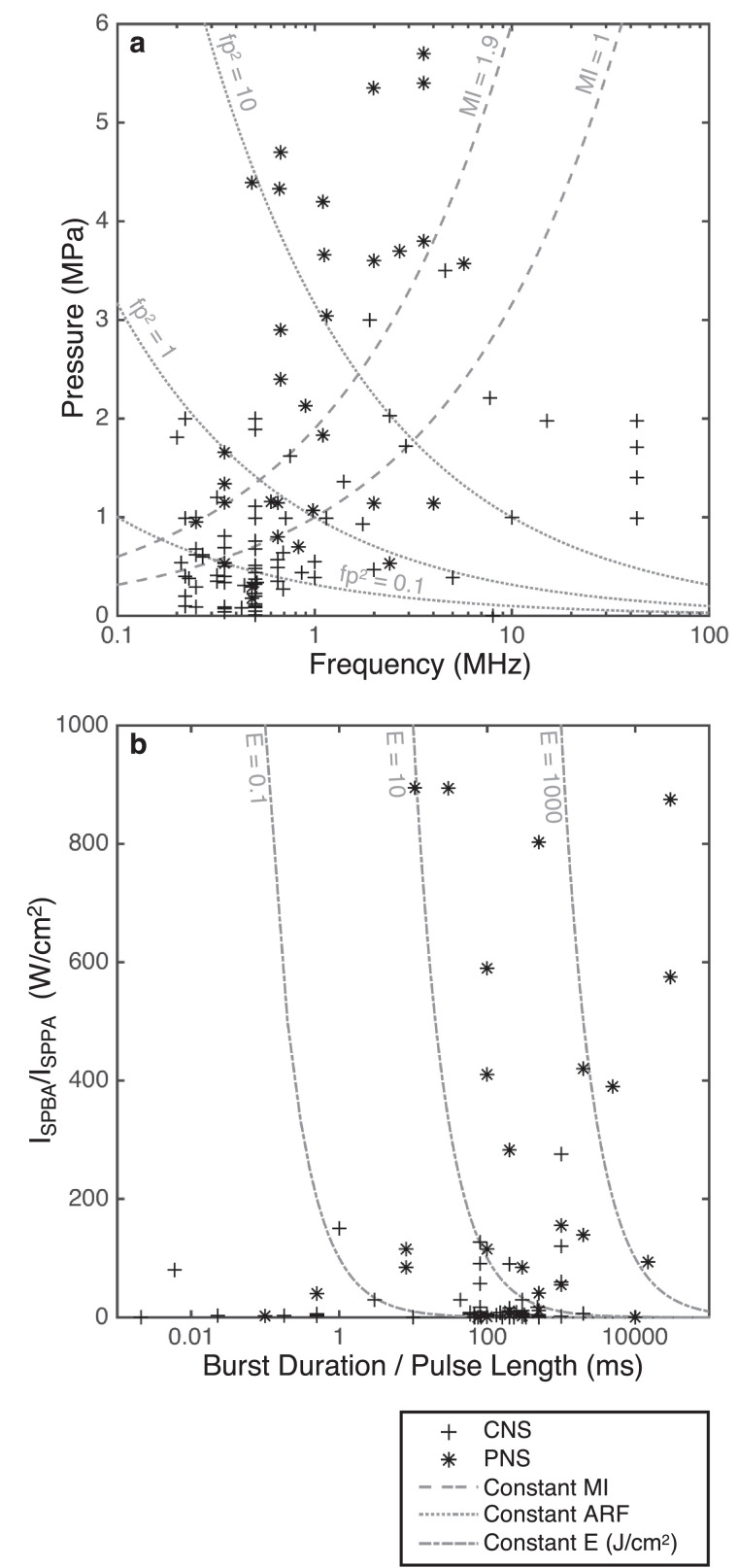
Comparison of parameters employed in the central nervous system (CNS, cross) and the peripheral nervous system (PNS, star). (a) Instantaneous peak pressure (*p*) against driving frequency (*f*), with lines of constant mechanical index (MI) and *fp*^2^, a measure of acoustic radiation force (ARF) (log scale). (b) For continuous wave: pulse-averaged intensity (I_SPPA_) against pulse length (PL). Lines of constant energy density (I_SPBA_ × BD or I_SPPA_ × PL) are also displayed (log scale). ARF = acoustic radiation force; BD = burst duration; CNS = central nervous system; E = energy density; *f* = driving frequency; *fp*^2^ = measure of acoustic radiation forces; I_SPBA_ = spatial-peak burst-averaged intensity; I_SPPA_ = spatial-peak pulse-averaged intensity; MI = constant mechanical index; *p* = instantaneous peak pressure; PL = pulse length; PNS = peripheral nervous system.

Establishing the safety of US in the brain is paramount to enabling FUS as a viable NIBS method. Many of the studies on ultrasonic neuromodulation have also published safety data relating to any damage, or the absence of damage, that occurred during the stimulation. The published data related to such safety concerns will be reviewed in this section, and existing published guidelines on the safety of US pulses will be examined. Fig. 2, Fig. 3, Fig. 4, Fig. 5 display the US parameter spaces utilized to date for neuromodulation in the CNS and PNS indicating where any histologic assessments have been conducted and if any damage was observed. These are discussed in more detail in the Review of Acoustic Parameters subsection.

### Central nervous system

The early work in hippocampal slices and mice conducted a series of assays to assess safety. In slices, repeated stimulation every 8 min for 36–48 h did not alter the structure of cell membranes (Tyler et al. 2008). There was no sign of BBB damage or changes in synaptic morphology, density or cortical neuropil structure (up to I_SPTA_ = 142.20 mW/cm^2^), no neurologic abnormalities during rotorod or wire-hanging tasks and no increase in the density of apoptotic neurons or glial cells (up to 300 mW/cm^2^) (Tufail et al. 2010).

Further histologic assessments, including hematoxylin and eosin staining and DNA fragmentation (TUNEL) assays, have been conducted following stimulation in a number of animal studies and revealed no damage as shown in Figures 2 and 3 (Dallapiazza et al., 2018, Mehić et al., 2014, Min et al., 2011a, Li et al., 2016, Lee et al., 2017, Yang et al., 2012, Yoo et al., 2011a). Work in disease models for neurodegenerative diseases has also not shown any damage due to low-intensity US in control groups as well as offered a neuroprotective benefit following toxin exposure, reducing oxidative stress (Zhao et al. 2017), myelin loss and apoptosis (Yang et al. 2015), resulting in increased cell viability.

Out of the 54 CNS studies reviewed, only two reported damage associated with US stimulation. In rats, one animal exhibited several areas containing hemosiderin, indicating the potential of local bleeding (Kim et al. 2014a). The parameters used were an US frequency of 0.35 MHz at an I_SPPA_ = 22.4 W/cm^2^, MI = 1.38 and BI = 2 s. The second study, in sheep, showed that repeated stimulations (more than 500 trials) delivered at short BIs of 1 s resulted in small micro-hemorrhages (Lee et al. 2016c). However, at 5 s BIs, no damage was observed. These studies highlight the need to design US sequences that are away from any possible damaging levels by limiting the maximum intensities used and ensuring sufficient rest periods between pulses.

In terms of mechanical safety concerns, to our knowledge, no CNS neuromodulation study has detected direct evidence of cavitation during US stimulation. At the pressures utilized in humans (<600 kPa), it is unlikely to lead to large blood vessel rupture in the absence of UCAs. Although limited data have been published on the cavitation thresholds in brain tissue, a study in sheep at 0.66 MHz with short, two-cycle pulses required peak negative pressures of 12.7 MPa to induce bubble nucleation (Gateau et al. 2011). Using 20 s duration pulses at 220 kHz, cavitation was detected for source powers of 300 W and above (Xu et al. 2015), which resulted in a detectable lesion. These waveforms also led to significant temperature rises (>10°C) and consequently are far removed from the parameters utilized in ultrasonic neuromodulation.

Whilst histology in humans has clearly not been viable, neurologic examinations and MRI follow ups have not reported any adverse findings in any of the human studies, up to an I_SPPA_ of 11.6 W/cm^2^ (Lee et al., 2015, Lee et al., 2016b, Legon et al., 2014). The most severe complication was a headache experienced by one patient, which quickly resolved (Hameroff et al. 2013).

### Peripheral nervous system

Typically, US intensities for PNS stimulation have been higher than those required for neuromodulation in the CNS. Reversible CAP effects have not been associated with any damage in animals or humans. However, following prolonged US exposure, nerve damage has been detected and is linked with irreversible activity suppression (Colucci et al., 2009, Foley et al., 2008, Lele, 1963). In *ex vivo* crab nerve preparations, afterdischarge was observed at high intensities, 230 W/cm^2^ at 0.67 MHz that resulted in reduced CAPs (Wright et al. 2017). This was associated with significant cavitational activity and therefore probably arose as a result of membrane rupture.

In mice, damage was only observed in a positive control group using very high pressures of 5.4 MPa at a 90% BDC, 0.5 s PL (Downs et al. 2018) at 3.57 MHz. For lower pressures (0.53 MPa) at 350 kHz, no tissue damage or BBB disruption was detected (Kim et al. 2012). No damage was detected in human fingertip vibrotactile experiments (Dickey et al., 2012, Lee et al., 2014). Again, any damage parameters are highlighted in Figure 4.

### Safety metrics

The safety of US pulses for diagnostic imaging has been extensively studied (Duck 2008). Three metrics are typically quoted to ensure the safety of the incident US pulse: intensity, thermal index (TI) and MI. The TI is a measure of heating within the tissue. It is defined as the ratio of the acoustic power (*W_p_*) to the power required to raise the tissue by 1°C (*W_deg_*). Related to this, the estimated temperature rises can be calculated from eqn (3), which is an approximation to the Pennes bioheat transfer equation (Pennes 1948).

(2) $$TI=\frac{W_{p}}{W_{\deg}}$$

(3) $$\frac{dT}{dt}=\frac{2\alpha I}{\rho_{0}c_{p}}≃\frac{\alpha_{0}}{\rho_{0}^{2}c_{0}c_{p}}fp^{2}$$

The MI is a measure of cavitation related to the degree of induced bubble activity and hence to the probability of mechanical damage occurring within the tissue (Apfel and Holland 1991). The MI is defined as the ratio of peak negative pressure in MPa to the square root of the frequency in MHz.

(4) $$MI=\frac{PNP}{\sqrt{f}}$$

It should be noted that the MI was originally developed for short imaging pulses in water and blood to assess the likelihood of cavitation, and so its applicability to longer pulses utilized for ultrasonic neuromodulation is unclear. Subsequent studies have examined the use of MI for longer US pulses utilized in ARF imaging, which suggest that a modification of the frequency exponent from 0.5 to 0.75 shows a better fit with theoretical data (Church et al. 2015). However, experimentally obtained cavitation thresholds in tissue are much higher than the theoretical predictions, which suggests that the assumption of optimally sized cavitation nuclei existing in tissue is incorrect (Church et al. 2015). For injected gas-encapsulated UCAs, an alternative measure, the cavitation index, has also been developed to predict cavitation activity and modifies the frequency exponent in the MI to one. The associated thresholds for UCA rupture and subharmonic cavitation emissions are consequently much lower (Bader and Holland 2013). Therefore, whilst the exact form of the index to predict cavitation in tissue for long US pulses is under debate, we will use the MI as a reference for discussing the mechanical safety of neuromodulation US sequences given that this is the parameter quoted in most of the US safety guidelines. It may be that keeping pressure amplitudes below the MI limit is unnecessarily restrictive for neuromodulation; however, until validated safety studies have been carried out, particularly in brain tissue, it seems unwise to exceed the diagnostic MI limit at present.

### Safety guidelines

The Food and Drug Administration (FDA) guidelines for diagnostic US imaging devices complying with the output display standard, are as follows: the I_SPTA_ must not exceed 720 mW/cm^2^, the I_SPPA_ must not exceed 190 W/cm^2^, the TI must not exceed 6 and the MI must not exceed 1.9 (Duck 2007). The British Medical Ultrasound Society also has a set of guidelines for diagnostic imaging: the TI must be less than or equal to 0.7 for unlimited time or less than 3 if the duration is less than 1 min. The MI should also be less than 0.7 if UCAs are used, as there is a risk of cavitation above this threshold (Apfel and Holland, 1991, Safety Group of the British Medical Ultrasound Society 2010).

### Review of acoustic parameters

Tables 2 and 3 list the studies in the literature where US was used for neuromodulation in the CNS and PNS, respectively, without any additional exogenous agents, highlighting the key findings along with any reported safety information. The literature search was carried out using the keywords “ultrasound” and “neuromodulation” or “brain stimulation” or “LIFU” on the PubMed, ScienceDirect and Google Scholar databases. Any studies that used additional agents (for example: UCAs or nano-particles) or caused thermal ablation were neglected. In total, 77 studies were identified that used US to modulate the nervous system. For each study, the acoustic parameters were extracted or, if not given, calculated from the quoted parameters where possible. The full US parameter sets can be found in the Supplementary Information.

**Table 2:** Ultrasonic neuromodulation central nervous system (CNS) studies

Study	Organism & target	Key findings	Safety
Legon et al. (2018b)	Human (M1)	Combined US and magnetic stimulation. US inhibits amplitude of single-pulse TMS-induced MEPs and reduces reaction times during stimulus response task.	–
Legon et al. (2018a)	Human (Thalamus)	Modulation of sub-cortical nuclei. Attenuation of P14 SEP amplitude. Reduction in performance of discrimination task.	–
Lee et al. (2016b)	Human (V1)	Phosphene perception. fMRI: activation of V1, visual pathways & cognitive processes. Modulation of VEPs.	Neurologic examination, MRI follow up (0, 2, 4 wk) and follow-up interviews (2 mo): no abnormal findings across all participants.
Lee et al. (2016a)	Human (S1, S2)	Elicitation of tactile sensations on hand and arm. Simultaneous S1/S2 stimulation.	No adverse changes or discomfort in mental/physical status across all individuals.
Ai et al. (2016)	Human (M1, S1, caudate)	fMRI responses in sensorimotor & caudate regions.	–
Lee et al. (2015)	Human (S1)	Elicitation of peripheral sensations on hand and arm. Modulation of SEPs.	Neurologic and neuroradiologic assessment did not show any safety concerns.
Mueller et al. (2014)	Human (S1)	Modulation of EEG dynamics, including phase and phase rate in beta and gamma bands.	–
Legon et al. (2014)	Human (S1)	Modulation of SEPs and alpha, beta and gamma frequency bands. Improvement in discrimination tasks.	–
Hameroff et al. (2013)*	Human (Posterior frontal cortex)	Improvement in mood scores. Small pain reduction but not significant.	One subject experienced a headache, which quickly resolved. No other side effects up to 4 mo after the study.
Kubanek et al. (2017)	Monkey (FEF)	US stimulation to left (right) FEF shifted animals’ choices to rightward (leftward) target.	No long term bias in animals choices after 8 d of stimulation of each region.
Wattiez et al. (2017)	Monkey (FEF)	Single neuron recordings in SEF: activity changes following US stimulation of FEF. ∼40% of neurons modulated.	–
Deffieux et al. (2013)	Monkey (FEF)	Increased latencies in antisaccade task.	Stimulation effect was transient (no significant effects observed on the following control trials).
Dallapiazza et al. (2018)	Pig (Thalamus)	Reversible suppression of SEPs. Selective activation of sub-nuclei within somatosensory thalamus.	Histology: no gross or microscopic tissue damage.
Daniels et al. (2018)	Pig (AC)	AEP suppression.	–
	Rat (Inferior colliculus region)	AEP suppression.	Histology (H&E): no damage. No sign of inflammatory response or structural changes. AEP amplitude recovery 1 h to 1 mo.
Lee et al. (2016c)	Sheep (SM1, V1)	SM1: EMG response of contralateral hind leg. V1: VEPs.	Histology: small microhemorrhage for repetitive stimulation (≥ 500 stimulations delivered at 1 s intervals). Damage not seen at longer ISIs. Post-sonication behavior normal.
Fisher and Gumenchuk (2018)	Mouse (Cortex)	Reduction in latency and increased Ca^2^+ response following electrical stimulation with US pre-treatment.	Histology: no changes in distribution of glial fibrillary acidic protein or evans blue – no neural injury or BBB opening.
Han et al. (2018)	Rat (Motor cortex) & Cell cultures	Response robustness increased with intensity and linked with shorter latencies. Ketamine reduced Ca^2+^ transients in dose-dependent manner by up to 82%.	Histology (H&E): no obvious damage, morphologic changes, tissue bleeding, or cytoplasmic swelling.
Guo et al. (2018)	Guinea Pig (Various including A1, S1)	US response due to indirect cochlear fluid pathway rather than direct activation. Similar activity in A1, SC1 recorded irrespective of target location. US-evoked activity eliminated by removal of cochlear fluid.	–
Sato et al. (2018)	Mouse (Visual cortex)	Widespread neural activation through indirect auditory mechanism. Contralateral visual cortex had similar response kinetics to targeted side, but auditory cortex showed contralateral bias. Chemical deafening greatly reduced motor outputs.	–
Gulick et al. (2017)	Rat (Motor cortex)	Long-term modulation of electrical stimulation: reduced hind limb responses. Direct motor response had 3 s refractory period.	No behavioral changes observed following stimulation.
Lee et al. (2017)	Rat	–	Histology (H&E, TUNEL assay): no cell necrosis.
Li et al. (2016)	Mouse (Motor cortex)	Increased specificity and decreased latencies at 5 MHz compared with 1 MHz.	Histology (H&E): no evidence of tissue bleeding or cell necrosis.
Kamimura et al., 2016, Kamimura et al., 2015)	Mouse (Motor & cognitive areas)	Limb movement and eyeball dilation.	Histology (H&E): no damage.
Darvas et al. (2016)	Rat	EEG signal at the frequency of the US PRF was induced along with demodulated activity in gamma & beta bands: potential use of US to tag deep regions for EEG-based mapping.	–
Yu et al. (2016)	Rat	Localization of induced brain activity using electrophysiologic source imaging.	–
Moore et al. (2015)	Mouse (Somatosensory cortex)	US and optogenetic responses have similar form for pyramidal neurons, but not interneurons, but amplitudes 10- to 20-fold lower for US.	–
Ye et al. (2015)	Mouse (Motor cortex)	Success rate decreases with frequency for given intensity. Focal spot size did not have consistent effect on success rates; most of the variance can be explained by frequency. Success strongly correlated with cavitation index and particle displacement but not ARF.	–
Kim et al. (2015)	Rat (Visual cortex)	VEP magnitude suppression/enhancement dependent on intensity and BD. Threshold intensity to elicit response.	–
King et al. (2014)	Mouse (Motor cortex)	Differences in EMG response (magnitude and latency) following rostral or caudal stimulation.	–
Mehić et al. (2014)	Rat (Motor cortex)	Comparison of planar, focused and modulated-focused source using 1.75 and 2.25 MHz to generate a 0.5 MHz difference frequency. Large variance in responses. Robustness of motor movement scaled with Ispta.	All histology samples showed no damage to brain tissue.
Younan et al. (2013)	Rat	Motor responses: tail, fore and hind limbs, eye, single whisker. Pressure threshold for response dependent on anesthesia levels. Rat skull distributes field across whole brain and introduces pressure hot spots due to reverberations.	No change in behavior or weight was observed.
King et al. (2013)	Mouse (Motor cortex)	EMG motor responses. Anesthesia levels important. CW as effective as pulsed US. All or nothing responses. Responses occur due to stimulus onset (within 30–100 ms). Required intensity increases with frequency. Success rate increases with PRF from 100–3000 Hz. Key variable appears to be integral of amplitude over a time interval of 50 to 150 ms.	–
Yang et al. (2012)	Rat (Thalamus)	Reduction in extracellular GABA for at least 2 h following sonication. No change in glutamate levels.	Histology showed no abnormal findings at either the focus or along the beam path.
Yoo et al. (2011a)	Rabbit (Somatomotor & visual areas)	Bimodal modulation: excitation of motor response and suppression of p30 VEP component. EEG signals confirmed by BOLD fMRI.	Histology did not reveal any tissue damage. No TUNEL positive apoptotic cells or VAF positive ischemic cells were found. No increase in gadolinium signal, suggesting no BBB disruption.
Yoo et al. (2011b)	Rat (Thalamus)	Reduction in anesthesia times following FUS (up to 20 min).	–
Min et al. (2011a)	Rat (Thalamus)	Reduction in EEG theta bursts after epileptic seizure induction.	Histology: no tissue damage (H&E) or DNA fragmentation (TUNEL).
Min et al. (2011b)	Rat (Thalamus)	Increase in extracellular dopamine and serotonin levels for at least 2 h post-sonication.	–
Tufail et al. (2010)	Mouse (Motor cortex & hippocampus)	Increased cortical spiking. TTX blocked US-evoked activity. Mean failure rate increased from 0.25–5 MHz. Lower frequencies & I_sppa_s give more robust EMG responses. Evoked potentials in hippocampus followed by 3 s afterdischarge containing gamma, sharp wave ripple oscillations and increase in spike frequency. Increase in BDNF.	No evidence of BBB opening. No change in density of apoptotic glial cells or neurons. No differences in synapse density or cortical neuropil ultra-structure. No neurologic abnormalities during rotorod and wire-hanging tasks.
Koroleva et al. (1986)	Rat (Cerebral cortex & hippocampus)	Direct current potential changes and spreading depression waves.	–
Ballantine et al. (1960)	Cat (Edinger-Westphal nucleus)	Temporary dilation of eye.	No lesions observed.
Fry et al. (1958)	Cat (LGN)	Reversible suppression of VEPs.	No histologically detectable lesions.
Prieto et al. (2018)	Cell cultures	Patch clamp recordings: activation of Piezol but not Na_V_1.2 through membrane stress as a result of acoustic streaming.	–
Kubanek et al. (2018)	*Caenorhabditis Elegans*	MEC-4, a pore-forming subunit expressed in touch receptor neurons required for US-evoked behaviors. TRP-4 response due to background genetic mutation. 50% BDC and 300–1000 Hz PRF produce optimal response rates.	–
Menz et al. (2017)	*in vitro*: Isolated salamander retina	US stimulation results in micron-scale displacements. Efficacy increased with frequency, consistent with an ARF-mediated mechanism.	–
Kim et al. (2017)	*in vitro*: Hippocampal slice	MEA: region and threshold-specific increased spike activity during and after US stimulation.	–
Menz et al. (2013)	*in vitro*: Isolated salamander retina	US evoked strong response similar to visual response but with shorter latencies. US activated other cells beyond photoreceptors. PRF 15 Hz to 1 MHz had no effect on responses; only temporal-averaged power important.	–
Choi et al. (2013)	*in vitro*: Rat hippocampal neurons	MEA: increased spiking and bursting. Effect observed post exposure. Largest firing rate at 0.8 MPa, decreased at higher pressures.	–
Tyler et al. (2008)	*in vitro*: Hippocampal slices and isolated mouse brain	US-induced APs during whole-cell current clamp recordings in CA1 pyramidal neurons. Triggering of voltage-gated Na^+^ and Ca^2+^ channels, vesicle exocytosis and synaptic transmission. Addition of TTX and Cd^2+^ blocked Na^+^ and Ca^2+^ transients, respectively.	Repeated stimulation (36–48 h) did not alter fine membrane structure.
Khraiche et al. (2008)	*in vitro*: Hippocampal slices	MEA: US can excite neurons and increase firing rates.	–
Bachtold et al. (1998)	*in vitro*: Hippocampal slices	Enhancement and depression of electrically evoked potentials.	–
Rinaldi et al. (1991)	*in vitro*: Hippocampal slices	Depression of electrically evoked potentials.	–

⁎ GE LOGIQe US scanner (GE Medical Systems, China) with 12 L-RS imaging probe. A1 = primary auditory cortex; AC = auditory cortex; AEP = auditory evoked potential; AP = action potential; ARF = acoustic radiation force; BBB = blood–brain barrier; BD = Burst duration; BDC = Burst duty cycle; BOLD = blood oxygen level dependent; CA1 = *Cornu Ammonis 1* (hippocampal subregion); CW = continuous wave; EEG = electroencephalography; EMG = electromyography; FEF = frontal eye field; fMRI = functional magnetic resonance imaging; GABA = γ-aminobutyric acid; H&E = hematoxylin and eosin (staining); I_SPPA_ = spatial-peak, pulse-averaged intensity; I_spta_= spatial-peak, temporal-averaged intensity; LGN = lateral geniculate nucleus; Ml = primary motor cortex; MEA = multi electrode array; MEC-4 = Mechanosensory protein 4 (ion channel subunit); MEP = motor evoked potential; MRI = magnetic resonance imaging; PRF = pulse repetition frequency; S1 = primary somatosensory cortex; S2 = secondary somatosensory cortex; SEF = supplementary eye field; SEP = somatosensory evoked potential; SM1 = primary sensorimotor area; TMS = transcranial magnetic simulation; TRP-4 = Transient receptor potential 4 (ion channel); TTX = tetrodotoxin; TUNEL =  Terminal deoxynucleotidyl transferase dUTP nick end (DNA fragmentation assay); US = ultrasound; V1 = primary visual cortex; VAF = Vanadium acid fuchsin (staining); VEP = visual evoked potential.

**Table 3:** Ultrasonic neuromodulation peripheral nervous system (PNS) studies

Study	Organism & target	Key findings	Safety
Lee et al. (2014)	Human (Fingertip)	Induction of different peripheral sensations (thermal, vibrotactile and nociception) depending on US parameters. CW did not induce sensations. Thermal responses maximum over a band of intensities (I_sppa_ = 10–30 Wcm^−2^), whereas for vibrotactile and nociception, response rate increased with intensity. Greater response rate at 350 kHz than 650 kHz.	No short-term or long-term tissue damage to insonified finger.
Legon et al. (2012)	Human (Fingertip)	US induced evoked potentials similar to other stimulus modalities. The waveform can be adjusted to preferentially stimulate different fibers (*Aβ, Aδ* and C) and the subsequent somatosensory neural circuits as confirmed by fMRI.	–
Dickey et al. (2012)	Human (Fingertip)	Sigmoidal response rate with increasing intensity. High specificity (participants ability to determine when US applied) indicates unique tactile sensations induced by US. Response correlates with density of mechanoreceptors.	No psychological or physiologic changes (assessed by questionnaire).
Gavrilov et al. (1977a)	Human (Hand, forearm)	Increasing intensity: Tactile, temperature and, finally, pain sensations. At deeper targets, only pain elicited. Longer stimuli (>100 ms), sensations present at start and end of waveform. Temperature sensations dependent on temperature of water bath that hand is immersed in. Cavitation detected before onset of pain sensations.	–
Downs et al. (2018)	Mouse (Sciatic nerve)	EMG activity and visible muscle activation for *p* > 3.2 MPa and BDC > 35%. A break period of 20–30 s improved the next stimulation success rate to 92%. Latencies similar to electrical stimulation.	Histology: no damage detected for successful US stimulation parameters or negative control groups. Damage observed for positive control (5.4 MPa, 90% BDC, 1 kHz PRF, 0.5 s BD) and for PL > 30 ms at 5.7 MPa.
Casella et al. (2017)	Rat (Posterior tibial nerve)	Inhibition of rhythmic bladder contractions. Longer latency and refractory periods compared with electrical stimulation.	–
Ni et al. (2016)	Rat (Sciatic nerve)	Improved regeneration and functional recovery following crush injury. BDNF levels increased for first 2 wk following treatment.	–
Juan et al. (2014)	Rat (Vagus nerve)	Decrease in electrically evoked CAPs; effect increased in magnitude with I_spta_. Decrease in conduction velocities.	–
Tych et al. (2013)	Rat (Sciatic nerve)	US threshold for paw withdrawal reduced for neuropathic tissue compared with sham surgery tissue.	–
Kim et al. (2012)	Rat (Abducens nerve)	Eyeball movement.	Histology (H&E, trypan blue): no damage or BBB disruption.
Foley et al. (2008)	Rat (Sciatic nerve)	Increased reduction in CMAPs with intensity. CMAP amplitude recovered by 28 d in all but highest intensity, which showed no recovery.	Histology: increased levels of damage as intensity increased up to complete axonal degeneration and necrosis.
Ellisman et al. (1987)	Rat (Dorsal nerve roots)	Electron microscope: morphologic changes in rats at myelination development stage (3–5 d old)—enlargement of periaxonal space, abnormal morphology of nodes of Ranvier and demyelination.	See results.
Gavrilov (1984)	Various	Human: skin receptors, threshold value dependent on density of receptors distributed on skin surface. Perception of 400 ms pulse the same as two spaced 10 ms pulses. Use of US for diagnosis of neurologic diseases based on tactile sensation response. Skate fish: stimulation of electroreceptors only achieved with pulsed US and not CW.	–
Gavrilov et al. (1977b)	Cat (Pacinian corpuscle), Frog (Ear labyrinth)	APs induced in Pacinian corpuscle for intensities in range 0.1–4.2 Wcm^−2^. Amplitude of receptor potentials increased with intensity. Evoked potentials in frog auditory brain at intensities as low as 0.01 W cm^−2^ similar in shape to sonic stimuli.	–
Lele (1963)	Cat, Monkey, Human, Earthworm.	Progressive US dose leads to initial AP amplitude enhancement, then reversible and finally irreversible depression. Conduction velocities increase with dose. Physiologic effects reproduced by heat application.	Enhancement/reversible depression: undistinguishable from unirradiated nerves. Irreversible depression: nodularity, fragmentation of axis cylinders restricted to irradiated section of nerve (indistinguishable from heat damage). Prolonged, intense US irradiation without rise in nerve surface temperature without apparent physiologic and anatomic effects.
Young and Henneman (1961)	Cat (Saphenous nerve)	Differential blocking of mammalian nerves. C-fibers most responsive. A-a least sensitive. Reversible and then permanent block with increasing US dose.	–
Wahab et al. (2012)	Earthworm (Giant Axon)	Cumulative ARF negatively correlated to reduction in conduction velocity and AP amplitude. At low impulses, enhancement in amplitude before dropping at longer exposure times. Final changes semi-permanent: no recovery within 15 min.	Semi-permanent effects in reduction of AP amplitudes following repeated single pulse sonications 100 times a second for over 200 s.
Wright et al., 2017, Wright et al., 2015)	*ex vivo*: Crab (Leg nerve axon)	Unpredictable responses with slight preference for first stimulus. Lowest intensity for successful stimulation was 100 Wcm^−2^ (1.8 MPa) at 0.67 MHz. No responses at 1.1 or 2 MHz. Cavitation signals detected for all successful stimuli; afterdischarge at 230 Wcm^−2^ resulting in reduced CAPs – probably due to cavitation-induced membrane rupture.	
Colucci et al. (2009)	*ex vivo*: Bullfrog (Sciatic nerve)	1.986 MHz: reduction in CAP amplitude, thermal effect matched by experiments varying water bath temperature. 0.661 MHz: discrepancy with thermal effects. Pulsed US: initial small increase in CAP then reduction.	Histology (H&E): 1.986 MHz, little or no damage consistent with thermal effects. 0.661 MHz, varying levels of damage depending on intensity. At higher intensities evidence of cavitation.
Tsui et al. (2005)	*ex vivo*: Bullfrog (Sciatic nerve)	Increased conduction velocity with power. Amplitude increased by 9% at 1 W but then decreased at higher powers.	–
Schelling et al. (1994)*	*ex vivo*: Frog (Sciatic nerve)	CAPs generated similar in shape but lower in amplitude than electrically induced CAPs. Movement away from the focus prevented CAP generation until air bubbles where added.	–
Mihran et al. (1990)	*ex vivo*: Frog (Sciatic nerve)	Latency of applied US results in different responses: enhancement or suppression of electrically induced CAP. Required BD to induce response reduced as intensity increases.	–
Fry et al. (1950)	*ex vivo*: Crayfish (Ventral nerve)	Increased spiking and then reversible depression of spontaneous activity.	–

⁎ Shock wave source. AP = action potential; ARF = acoustic radiation force; BBB= blood–brain barrier; BD = burst duration; BDC = burst duty cycle; BDNF = brain-derived neurotrophic factor; CAP = compound action potential; CMAP = compound muscle action potential; CW = continuous wave; EMG = electromyography; fMRI = functional magnetic resonance imaging; I_spta_= spatial-peak, temporal-averaged intensity; H&E = hematoxylin and eosin (staining); p = pressure (peak instantaneous); PL = pulse length; PRF = pulse repetition frequency.

Figure 2 displays the peak instantaneous pressure against driving frequency for all of the CNS studies. Here, only the maximum pressure used in each experiment or study has been extracted. For studies that indicate damage, the minimum pressure at which damage was reported are plotted in order to focus on safety. Successful stimulation may therefore have occurred at lower thresholds. The data have been split into four categories: human, large animal, small animal and *in vitro* work. Where histologic analysis has been conducted, the markers are filled with blue, indicating no damage, or red to indicate damage. Contours lines of MI = 1.0 and MI = 1.9 are plotted in order to place data relative to the safety metrics. Also shown are lines of constant *fp*^2^, which correspond to a constant ARF, as shown in eqn (1). It is noted that this expression takes a similar form to the heating rate $\left(\frac{dT}{dt}\right)$, as shown in eqn (3), where *c_p_* is the specific heat capacity of the tissue. For *fp*^2^ = 1, this results in a heating rate of approximately 1°C per second. Figure 2a shows the data on a logarithmic frequency scale spanning almost three orders of magnitude. The majority of the studies have been conducted in the sub-megahertz region, typically at pressures below 1 MPa. This subset of the full parameter space also corresponds to the likely range of viable parameters for transcranial human applicability from both a delivery and ultimately a safety perspective. It can be seen that the human studies fall within the FDA US imaging guidelines, with an MI of below 1.9 and an I_SPPA_ below 190 W/cm^2^ (the exact values can be found in the Supplementary Information). This region is zoomed in on in Figure 2b. It is noticeable that the two reports of damage occur in the top left quadrant of the plot with pressures over 0.65 MPa at the lower end of the frequency spectrum (250–350 kHz) and is thus correlated with higher MI values. Although it is also noted that there have been studies both in a large animals and small animals that report no damage at similar frequencies but at higher pressures, up to 1.2 MPa.

The same data but plotted as the I_SPBA_ versus BD (burst wave) or I_SPPA_ versus PL (continuous wave) are shown in Figure 3. Contours of constant energy per unit area are also displayed, that is, either I_SPBA_ × BD or I_SPPA_ × PL. The wide span of different parameters is again evident in Figure 3a with PLs ranging from hundreds of nanoseconds to the order of seconds. The majority of the data are clumped in one region between 20 and 550 ms and at intensities less than 30 W/cm^2^, which is shown in more detail in Figure 3b. On this axis, the damage parameters are slightly separated from those that reported no damage and are correlated with higher energy density levels. Unfortunately, in general, insufficient data were available to calculate the total cumulative energy (energy delivered in one burst [pulse] multiplied by the total number of bursts [pulses]) for each study to calculate a total energy dose. This may have further separated the data given that in the sheep study only repeated application of more than 500 trials resulted in microhemorrhage (Lee et al. 2016c). That said, the other report of damage only applied three repeated trials but with a higher energy density per burst (Kim et al. 2014b).

For the PNS, the data do not exhibit clusters as for the CNS literature. For both plots, pressure versus frequency (Fig. 4a) and intensity versus PL (Fig. 4b), large variations in the acoustic parameters between different studies are observed. There are also more reports of damage following the trend described in the Peripheral Nervous System section, where initially reversible effects without damage are observed, followed by irreversible changes linked with nerve damage as more energy was deposited. However, again, insufficient data were available in order to plot the data against a total cumulative energy measure. Some of the irreversible effects are likely due to thermal damage as a consequence of the higher intensities and longer exposures used in PNS stimulation, but for shorter pulses, cavitation has also been linked to damage through nerve rupture (Wright et al. 2017).

Comparing the CNS and PNS parameters, it can be seen that the pressures and intensities used for PNS modulation tend to be higher than for the CNS, as shown in Figure 5. Whilst these plots only depict the maximum parameters, a similar trend is also observed for success thresholds, indicating more energy is required to elicit responses in peripheral nerves. This may be attributed to numerous factors, including differences in cell morphology, axonal bundle sizes and nerve myelination, and therefore potentially requiring different mechanisms to stimulate them.

### Thermal effects

Thermal effects are clearly involved in some of the peripheral nervous studies with effects replicated in temperature-controlled water baths (Lele 1963), and temperature changes can affect neural function as described in the Thermal Effects Mechanism section. However, for most of the recent brain studies, expected temperature rises are typically less than a 10th of a degree (Lee et al., 2016b, Lee et al., 2016c, Khraiche et al., 2008, Tufail et al., 2010), which should be negligible. Consequently thermal effects are not considered to contribute to neurostimulation responses.

Retrospective temperature simulations in several rodent setups (Constans et al. 2018) revealed that in general, temperature effects could be neglected, but in one study (Kamimura et al. 2016), a temperature rise of 7°C was reported as a result of thermal diffusion from the skull bone into the brain. Therefore, care must be taken to ensure off-target temperature rises do not occur through skull-induced heating, potentially resulting in thermal modulation.

## Delivery

A further challenge to implementing US as a neuromodulatory tool in the CNS is the delivery of US through the cranium. Skull bone is a highly heterogeneous structure that has both a higher density and sound speed, resulting in a large impedance mismatch with respect to the soft tissue that surrounds it. Moreover, it is a multi-layered structure with a hard cortical shell and a blood- and fat-filled, inner cancellous bone layer. The trabecular structure of this internal layer results in strong scattering of the acoustic wave at frequencies above 1 MHz (Pinton et al. 2010), effectively making the skull a low pass frequency filter, thereby limiting the viable frequency range for transcranial US propagation. The presence of hair may also increase losses; a study in an *ex vivo* cadaver model reported a temperature elevation drop of 17% at 710 kHz but negligible losses at 220 kHz due the addition of human hair (Eames et al. 2014).

In rodents, thin skull bone allows US to easily penetrate into the brain, but standing waves can be formed from reflections off the opposite side of the skull, leading to complex fields and pressure hotspots away from the target location (Constans et al., 2017, Younan et al., 2013). In scaling up from small animals to large animals and humans, the thicker skull presents different challenges: primarily increased attenuation and aberration of the acoustic wave (Fry and Barger, 1978, White et al., 1978), shifting the focus inside the brain.

The development of multi-element arrays featuring hundreds to thousands of individual transducers (Hynynen et al., 2004, Hynynen et al., 2006) has enabled correction of the US wave to compensate for the aberrations and effectively focus within the brain to deep targets. These arrays have been primarily implemented for thermal ablation applications, allowing for treatment of brain tumors (McDannold et al., 2010, Ram et al., 2006), neuropathic pain (Jeanmonod et al. 2012), obsessive-compulsive disorder (Jung et al. 2015), essential tremor (Chang et al., 2014, Elias et al., 2013) and Parkinson's disease (Magara et al. 2014).

However, transducer arrays are highly complex as well as expensive thus making them prohibitory to the majority of research groups in the early stages of research into US-mediated neuromodulation. Moreover, with many of the initial brain targets for probing the effects of US-induced brain stimulation situated close to the cortical surface (Lee et al., 2016b, Legon et al., 2014), arrays may not be required to treat these areas, especially given the fact that stimulation has been shown to be more robust at lower frequencies (King et al., 2013, Tufail et al., 2010, Ye et al., 2015), which are less subject to distortion during transcranial propagation. Consequently, more simplistic transducer configurations can be utilized to focus to peripheral brain targets at a much reduced cost. Computational approaches can be effective here in order to determine the efficacy of targeting the brain with these simpler source conditions, such as single-element transducers, specifically for neuromodulation (Constans et al., 2017, Mueller et al., 2017, Robertson et al., 2017).

However, some targets are likely to remain difficult to access *via* single-element transducers owing to the heterogeneous skull structure overlying their location. For these locations, the development of an acoustic lens could correct for the induced aberrations and reform the acoustic focus over the intended target (Maimbourg et al. 2018). The lens has a variable thickness to adjust the phase of the incident wave based on the sound speed of the material. Consequently, a single-element transducer can be turned into an effective array, enabling the treatment of previously intractable sites. Although the lens is therefore individual and target specific, it can be constructed at a fraction of the cost of an array, and for repeated treatments, as may be the case for clinical applications, it should not need further adjustment once it has been created.

In summary, whilst arrays are likely to be required to pinpoint specific deep-seated CNS brain targets, single-element transducers may be viable for targeting the cortical surface with lenses, providing a solution for intermediate locations. In the PNS, the complications associated with the skull are avoided, but the depth of the target nerve and the presence of bone and air in the acoustic path could also require a complex US source.

## Monitoring

Whilst numerical modelling is an invaluable tool in determining the required source conditions for transcranial targeting, the ability to monitor where the US is being delivered would enable confirmation that the intended brain target is being stimulated. For thermal ablation applications, low-powered sonications raising the tissue by a few degrees can be utilized in combination with magnetic resonance (MR) thermometry (Rieke and Pauly 2008) to determine the US focus. However, for neuromodulation applications where thermal rises are estimated to be negligible in the majority of cases, this technique is unviable for monitoring purposes. Indeed, MR thermometry did not detect any temperature changes for frequencies between 220 kHz and 1.145 MHz up to pressures of 1 MPa (Dallapiazza et al. 2018).

MR ARF imaging offers an alternative form of monitoring with tissue displacements encoded as phase shifts (Kaye and Pauly 2014). However, this technique is still under development and typically requires higher pressures than those needed to induce neuromodulation, albeit at shorter PLs, in order to image the subsequent tissue displacements (Hersh et al., 2018, Liu et al., 2015). Therefore, the safety of this approach for brain applications must also be established. Consequently, there is still a need to develop monitoring techniques for non-thermal US pulse sequences.

In terms of monitoring brain activity, EEG and fMRI are the cornerstones of non-invasive techniques. EEG uses multiple electrodes placed on the scalp to measure evoked potentials but is relatively superficial, making it difficult to monitor the activity of deep-seated targets. One group have explored tagging deep brain volumes using a unique, high-frequency electrical signal generated by US above the normal frequency range for neural firing rates, thus extending the use of EEG to deeper brain targets (Darvas et al. 2016). fMRI approaches are more applicable to measuring brain activity at deep targets with 3-D spatial mapping as well as for investigating connectivity between different brain areas. This makes for a powerful tool for monitoring real-time US-induced brain activity as well as longer term connectivity effects, but there are also concerns that acoustic pressure-mediated mechano-vascular coupling may also give rise to BOLD signals (Lee et al. 2016b) and therefore needs further investigation.

As an alternative to fMRI, functional US imaging (Macé et al. 2011) may prove to be a useful modality for imaging microvascular dynamics in the brains of small animals at a superior temporal resolution, but for now the technique is not scalable to large animals and humans because of the increased skull thicknesses.

## Discussion

In this review, we have summarized the work of the past several decades, demonstrating the ability of US to modulate neural activity in both the central and peripheral nervous systems. US has been shown to evoke a response in a wide range of neuronal targets including cell cultures, hippocampal slices, small animals, large animals and now nine reports in humans. These data provide a wealth of information as to how neural systems can be stimulated by US over a range of length scales with a multitude of methodologies and techniques employed.

At the cell level, fluorescence imaging and patch clamp recordings showed transient Na^+^ and Ca^2+^ ion currents in response to US. These ionic currents likely result from the incident US energy interacting with cell membranes; potentially through conformational changes in the lipids in the membrane or by mechanosensitive ion channels. The US stimulation has been shown to result in both excitatory and inhibitory responses in hippocampal slices. Two mechanisms, ARF and cavitation, are plausible at a biophysical level to produce neurostimulation and perhaps both can play a role, depending on the acoustic parameters and cell types. However, at the pressures employed in the reviewed human trials (<650 kPa), the likelihood of cavitation is very low.

In rodents, US has been shown to affect both the motor cortex and the visual system. In the latter case, VEPs could be suppressed or enhanced, consistent with what was observed at a cell level. For large animals, evoked potentials have been reported in the motor and visual pathways, and in non-human primates, modulation of latencies in the visual system confirmed the ability to modulate high-level cognitive behavior. In humans, stimulation of the visual system has resulted in phosphenes, the somatosensory system in sensations felt in the hands and the motor cortex in the suppression of MEPs.

There is now a body of evidence, which runs the gamut from isolated cells to cognitive responses in humans, that US does result in neurostimulation. The data show that US has the potential to enhance or suppress nerve firing depending on the US parameters, although at the network level, whether a stimulation results in excitatory or inhibitory behavioral outcomes will also depend on the connectivity of the neurons. The ability to use low intensity focused US for non-invasive, anatomically precise neuromodulation in humans has thus generated much excitement because of its potential both for understanding normal brain function and for diagnostic and therapeutic applications in disease. Proposed areas of clinical utility include treatment of neuropsychiatric disease (Tsai 2015), suppression of epileptic activity (Min et al. 2011a), temporary blockade of peripheral nerves involved in pain signaling (Downs et al. 2018) and pre-surgical verification of CNS targets for ablation. However, many mechanistic questions remain to be answered.

An area of caution is the potential to directly activate the auditory cortex and higher order processes *via* US (Guo et al., 2018, Sato et al., 2018). These studies motivate careful attention to sham and control groups when designing trials to ensure that electrophysiologic and behavioral outcomes are not confounded by additional auditory activation and are a result of stimulation of the intended target. However, one should also note that auditory-mediated effects are very unlikely to explain modulation of saccadic behavior changes in neuronal activity recorded in the medial frontal cortex following US over the FEF (Deffieux et al., 2013, Wattiez et al., 2017). Related to this is the ability to focus to the desired target region. Historically, the skull has been considered an impossible barrier for US; however, at sub-megahertz frequencies, it is possible to focus US into the brain. For many peripheral brain targets, relatively inexpensive and simple single-element transducers can be employed, but for deeper targets or those underlying strongly heterogeneous skull bone, more sophisticated strategies will be required, for example, a custom lens or an US array.

Another consideration that is often overlooked in mechanistic studies using *in vitro* setups is that the local environment may be very different from that experienced by neurons *in vivo*; both biologically, in that cells are in artificial environments, but also because of acoustically reflective surfaces (*e.g.,* coverslips, patch pipettes and air interfaces), the acoustic field and hence stimulus, will be affected. Accordingly, understanding the limitations of such artifacts associated with the *in vitro* system is something that should be appreciated when interpreting the data.

The US parameters that the community seems to be settling on for CNS stimulation are a frequency of approximately 200–500 kHz delivered as a 300–500 ms burst of about 0.5 ms pulses at a PRF of about 1 kHz, with pressure amplitudes on the order of 0.1–0.6 MPa in the brain. We carried out an analysis of the safety metrics developed for diagnostic US imaging for the US parameters used in the brain and found that the US parameters employed in the human trials would be considered safe from an imaging regulatory view. However, we note that the pulsing paradigm used in US stimulation studies involves longer pulses than used in US imaging and this may have an impact on cavitation thresholds, which should be considered further. We anticipate that further work in the safety area could allow a larger range of acoustic parameters to be employed than has been used to date. We have identified the key US parameters that would be helpful for researchers to report so that relevant mechanistic or safety metrics can be calculated.

In conclusion, US is emerging as an exciting NIBS method that has been demonstrated to safely and reversibly modulate the CNS. In the PNS, the intensities used are generally higher, and this may explain why more damage has been reported. More work is needed to fully explore the US parameter space from both a safety and a mechanistic perspective and to develop methods in which US can be robustly targeted to different regions within the brain. One goal for the community would be to develop appropriate guidelines for the use of ultrasonic neuromodulation in both research and clinical settings, as has been done for TMS (Rossi et al. 2009) and tCDS (Nitsche et al. 2008). However, even with the current understanding, US neuromodulation is a potent tool for basic neuroscientific research as well as a promising clinical utility.

## Appendix A Ultrasound parameter nomenclature

In our experience of writing this review and collating the US parameters from the diverse literature set, we found some ambiguities and differences in the naming protocols for the US sequences. We have therefore attempted to introduce a consistent naming convention as defined in the Ultrasound Exposure section. The confusion surrounding the parameter definitions may stem from fundamental differences in conventions from scientists with either an US or brain stimulation (TMS) background. For example, in the US community, a short, continuous wave pulse is often referred to as a tone burst, whereas in the TMS literature, a burst would refer to multiple discrete pulses. We have therefore chosen to refer to a short, continuous wave US waveform as a pulse. A burst is then made up of multiple repeated pulses at a specified PRF. The intensities are then defined with respect to the averaging over one pulse, burst, or the total experimental time.

A similar confusion is also observed for the ISI. Again, the TMS convention defines the ISI as the time between the end of one burst and start of the next. That is, the cooling period between bursts rather than the inverse of the BRF. Here, we have defined the BI as the inverse of the BRF.

We also recommend that future neuromodulation studies should quote sufficient parameters in order to fully define the US sequences and intensities. At a minimum, *f, p_max_*, I_SPPA_, PL, PRF, BD and ISI should be quoted, along with the relevant timescales over which the intensities are averaged.

## Appendix B Supplementary materials

Image, application 1
