# Supplementary material for: Ultrasound Neuromodulation: A Review of Results, Mechanisms and Safety
Source: Ultrasound Med Biol. 2019 Jul;45(7):1509–36. doi: 10.1016/j.ultrasmedbio.2018.12.015 (PMC6996285; doi:10.1016/j.ultrasmedbio.2018.12.015)
Supplement: Supplementary file 1 [file mmc1.pdf]

Ultrasound neuromodulation; a review of results, mechanisms and safety: Supplementary Information

J. Blackmore, S. Shrivastava, J. Sallet, C.R. Butler & R.O. Cleveland

November 27, 2018

This document contains the tabulated data extracted from the CNS and PNS ultrasonic neuromodulation studies, respectively. Where possible, parameters were directly taken from the literature and these are marked in the tables in black. If additional parameters could be calculated from the published parameters, this was done and these parameters are marked in red.

The majority of calculations where converting between intensities and pressures. We did this using the following equation assuming a plane time harmonic wave,

$$I = \frac{p^2}{2\rho_0 c_0}, \tag{1}$$

where p is the pressure amplitude, ρ_0 was taken to be 1046 kg/m^3 (Hasgall et al., 2015) and c_0 was 1562 m/s (Duck, 1990). We also converted between intensities over different time scales (temporal-averaged, burst-averaged and pulse-averaged) using the duty cycle information given in the reference.

Studies have been split into multiple rows where necessary in order to account for different experiments and ultrasound sequences.

If continuous wave ultrasound was used, the burst parameters are undefined and therefore marked with a dash.

References

Hasgall PA, Di Gennaro F, Baumgartner C, Neufeld E, Gosselin MC, Payne D, Klingenbck A, Kuster N. ITIS Database for thermal and electromagnetic parameters of biological tissues. Version 3.0, www.itis.ethz.ch/database.

Duck, FA. Physical properties of tissue: A comprehensive reference book. 1990;73–124.

Study	Category	Organism	f (MHz)	I_SPTA (mW/cm2)	I_SPBA (W/cm2)	I_SPPA (W/cm2)	p (MPa)	BD (ms)	BI (s)	PL (ms)	PRF (Hz)	BDC (%)	TDC (%)	N	TL (s)	MI	Histology	Damage	Notes
Legon et al., 2018	H	Human (M1)	0.5		0.16	0.44	0.12	500	8-10	0.36	1000	36		100					
Legon et al., 2018	H	Human (Thalamus)	0.5		2.53	7.03	0.48	500	4	0.36	1000	36		300		0.56			
Lee et al., 2016a	H	Human (V1)	0.27		0.85-5.8	1.7-11.6	0.24-0.62	300	2.5, 13	1	500	50		50		0.2-1.2			
Lee et al., 2016b	H	Human (S1, S2)	0.21		3.5-4.4	7-8.8	0.48-0.54	500	7	1	500	50						×	Dual single-element transducers.
Ai et al., 2016	H	Human (S1)	0.5		2.16	6	0.44	500	12.0-14	0.36	1000	36		90				×	
	H	Human(S1)	0.86		3	6	0.44	500		0.5	1000	50						×	
Lee et al., 2015	H	Human (S1)	0.25		0.25-1.25	0.5-2.5	0.13-0.29	300	3	1	500	50						×	
Mueller et al., 2014	H	Human (S1)	0.5		2.12	5.9	0.44	500	6	0.36	1000	36		120				×	
Legon et al., 2014	H	Human (S1)	0.5		2.12	5.9	0.44	500	6	0.36	1000	36		120				×	
Hameroff et al., 2013	H	Human (Posterior Frontal Cortex)	8	152		8.00E-06	5.00E-04								15	0.7		×	LOGIQe imaging probe.p, ISPPA estimated from MI value, derating and accounting for skull.
Kubanek et al., 2017	L	Monkey (Frontal Eye Field)	0.27				0.6	300										×	
Wattiez et al., 2017	L	Monkey (Frontal Eye Field)	0.32		-	1.9,5.6	0.24,0.41	-		100		-		386, 400				×	
Deffieux et al., 2013	L	Monkey (Frontal Eye Field)	0.32	13.5	-	4	0.35	-		100		-				0.6		×	
Dallapiazza et al., 2016	L	Pigs (Thalamus)	0.22		-	30	0.99	-		43.7	10	-			40		✓	×	Assuming ISPPA = ISA.
	L	Pigs (Thalamus)	0.71		-	30	0.99	-		43.7	10	-			40		✓	×	Assuming ISPPA = ISA.
	L	Pigs (Thalamus)	1.145		-	30	0.99	-		43.7	10	-			40		✓	×	Assuming ISPPA = ISA.
Daniels et al., 2018	L	Pig	0.23		-	4.6	0.38	-	3	100		-			52				Quoted pressures do not correspond with intensities. Assumed quoted intensity correct.
	S	Rat	0.23		-	2.3-4.6	0.27-0.38	-	3	100		-			52		✓	×	Quoted pressures do not correspond with intensities. Assumed quoted intensity correct.
Lee et al., 2016	L	Sheep (SM1)	0.25		1.7-5.9	3.4-11.8	0.33-0.62	300	5	1	500	50		100			✓	×	
	L	Sheep (V1)	0.25		0.85-7.15	1.7-14.3	0.24-0.68	300	1	1	500	50		100-600			✓	✓	Damage >500 times every second at moderate (ISPPA 6.6–10.5 W/cm2, MI=0.9–1.2).
Fisher et al., 2018	S	Mouse (Cortex)	0.51			0.69-3.5	0.17	1000		0.5	1000	50					✓	×	
Han et al., 2017	S	Rat (Motor Cortex)	0.35		0.4-4.67	1.16-13.55	0.06-0.69	66.67	2	0.23	1500	34.5							
	S	Rat (Motor Cortex)	0.35		1.19	3.46	0.34	66.67	2	0.23	1500	34.5			20 min		✓	×	
	I	Cortical cell cultures	0.35		0.02	0.056	0.04	66.67	2	0.23	1500	34.5							
Guo et al., 2017	S	Guinea Pig (A1, S1)	0.22			0.33	0.1	500		0.1-10									A1 activation.
	S	Guinea Pig (A1, S1)	0.22		0.73	1.22	0.2			0.1-10	10-1500								First spike latency experiment.
	S	Guinea Pig (A1, S1)	0.22			122	2	10											Auditory nerve transection.
	S	Guinea Pig (A1, S1)	0.22			1.22	0.2	1-6 s		0.5	1000-16000								Indirect somatosensory activation.
	S	Guinea Pig (A1, S1)	0.22			4.89	0.05-0.4	0.5-6 s		0.5-10									Cochlear fluid removed.
Sato et al., 2017	S	Mouse (Visual Cortex)	0.5		0.034-4.2	14	0.68	80		0.2	1500	30							
Gulick et al., 2017	S	Rat (Motor Cortex)	0.2	4500	30	60 (ISPTP = 100)	1.81	300	1-10	0.5	1000	50				3.1		×	US/ECS interaction. ISPPA calculated from quoted table.
	S	Rat (Motor Cortex)	0.2	9	30	60 (ISPTP = 100)	1.81	3	10	0.5	1000	50				3.1		×	EMG latency. ISPPA calculated from quoted table.
Lee et al., 2017	S	Rat	0.25			0.5-11	0.1-1										✓	×	
Li et al., 2016	S	Mouse (Motor Cortex)	1	130-230	2.3	4.6	0.39	300	3	0.5	1000	50	5		60		✓	×	
	S	Mouse (Motor Cortex)	5	130-230	2.3	4.6	0.39	300	3	0.5	1000	50	5		60		✓	×	
Kamimura et al., 2016	S	Mouse (Motor Cortex & Cognitive Areas)	1.9		44.1-275.4	22-137.7	1.2-3	1000	2	1	1000	50		10	20		✓	×	
Darvas et al., 2016	S	Rat	2		1.4	6.8	0.47	1000	2	0.2	1050	21		100	200				Assuming quoted ISPTA is averaged over the burst.
Yu et al., 2016	S	Rat	0.5		0.1-0.6 mW/cm2	0.7-4.6 mW/cm2	18.3-45.9 kPa	5-200	3	0.07	2000	14							38-252 mW/cm2 ISPTP, ring down pulse.
Moore et al., 2015	S	Mouse	0.35		0.03-0.1	0.07-0.23	0.09	10	0.14	0.214	2000	42.8			10 min				
Ye et al., 2015	S	Mouse (Motor Cortex)	0.3,0.4,0.5,0.6		-	0.1-3.5	0.06-0.34	-		66.7-133.3		-							A (planar)
	S	Mouse (Motor Cortex)	0.6,1,1.4		-	0.02-9.4	0.03-0.55	-		80		-							B
	S	Mouse (Motor Cortex)	1.4, 1.9, 2.4, 2.9		-	1.1-127	0.19-2.03	-		80		-							C
	S	Mouse (Motor Cortex)	0.3,0.4,0.5,0.6		-	0.04-3.1	0.04-0.32	-		80		-							D
	S	Mouse (Motor Cortex)	1.4		-	56.9	1.36	-		80		-							E
	S	Mouse (Motor Cortex)	2.9		-	90.7	1.72	-		80		-							E
Kim et al., 2015	S	Rat (Visual Cortex)	0.35	50-250	-	1-5	0.4	-		0.5	20-166	-	1-8.3		150				
King et al., 2014	S	Mouse (Motor Cortex)	0.5		-	3	0.2	-		80		-							
Kim et al., 2014a	S	Rat (Motor Cortex)	0.35		2.45-11.2	4.91-22.4	0.4-0.81	200-400	2	0.25-5	60-2800	30-70			1.38		✓	✓	Damage at 22.4 W/cm2 ISPPA, 300 ms, 3 bursts.
	S	Rat (Motor Cortex)	0.35		-	7.73	0.5	-		150		-					✓	×	
	S	Rat (Motor Cortex)	0.65		3.5-5	7-10	0.48-0.57	300		0.5-5	100-1000	50					✓	×	
Kim et al., 2014b	S	Rat (Thalamus)	0.35		3.5-4.5	7-9	0.48-0.54	300	2	0.5	1000	50			40 min				
	S	Rat (Thalamus)	0.35		3.5-4.5			300		0.25-5		30-70			40 min				PL and PDC variation.
Mehic et al., 2014	S	Rat (Motor Cortex)	0.5		0.15 - 5.25	0.5-17.5	0.13-0.76	58.7	1	0.2	1500	30		10	10		✓	×	Planar transducer.
	S	Rat (Motor Cortex)	1.75 & 2.25		1-8	3.3-26.7	0.18-0.93	14.7-58.7		0.1-0.2	1500	15-30		10	10		✓	×	mFU: two frequencies separated by 500 kHz.
Younan et al., 2013	S	Rat (Motor Cortex)	0.32		8.75	17.5	1.2	250		0.23	2000	50				2.2		×	Incorporates correction factor for standing waves.
King et al., 2013	S	Mouse (Motor Cortex)	0.5		-	0-16.8	0-0.51	-	>5	80		-							Acoustic intensity sweep.
	S	Mouse (Motor Cortex)	0.5		-	4.2	0.37	-		0-320		-							Sonication duration.
	S	Mouse (Motor Cortex)	0.25-0.6		-			-		40,000 cycles		-							Frequency sweep.
	S	Mouse (Motor Cortex)	0.5		23.7	0-79.02	0.03-1.11			0.2	1500								Pulsed v. CW.
	S	Mouse (Motor Cortex)	0.5		8.97	4.2-29.9	0.37-0.99	80		0.2	11-3000	30							Pulsed.
	S	Mouse (Motor Cortex)	0.5		-	0-16.8	0-0.51	-		0-480		-							Full CW parameter sweep.
Yang et al., 2012	S	Rat (Thalamus)	0.65	175	-	3.5	0.35	-		0.5	100	-			20 min		✓	×	
Yoo et al., 2011a	S	Rabbit (Somatomotor & Visual Areas)	0.69		1.6-6.3	3.3-12.6	0.64	0.5-2		0.05-50	10-1000	50					✓	×	
Yoo et al., 2011b	S	Rat (Thalamus)	0.65	300	-	6	0.49	-		0.5	100	-			20 min	0.61			
Min et al., 2011a	S	Rat (Thalamus)	0.69	130	-	2.6	0.27	-		0.5	100	-	5		2x3 min	0.33	✓	×	
Min et al., 2011b	S	Rat (Thalamus)	0.65	175	-	3.5	0.35	-		0.5	100	-	5		20 min	0.2			
Tufail et al., 2010	S	Mouse (Motor Cortex)	0.35		0.0362			229			1500								Initial LFP recordings and MUA in M1.
	S	Mouse (Motor Cortex)	0.5		0.06453	0.228	0.086	160			1500								Bilateral M1 stimuation.
	S	Mouse (Motor Cortex)	0.25		0.061-0.123	0.242	0.089	53-83		0.32-0.48	1200-1500	38.4-72				0.18			M1 parameter sweep.
	S	Mouse (Motor Cortex)	0.35		0.021-0.066	0.159	0.072	27-83		0.229-0.571	1200-2500	27.5-85.7				0.12			M1 parameter sweep.
	S	Mouse (Motor Cortex)	0.425		0.029-0.075	0.211	0.083	50-333		0.188-0.235	1500-3000	28.2-70.5				0.13			M1 parameter sweep.
	S	Mouse (Motor Cortex)	0.5		0.041-0.163	0.259-0.288	0.092-0.097	27-83		0.16-0.45	1200-2500	19.2-75				0.13-0.14			M1 parameter sweep.
	S	Mouse (Motor Cortex)	0.5		0.142-0.3			67.2	10	0.45	1500				30 min		✓	×	BBB disruption check.
	S	Mouse (Hippocampus)	0.25		0.084					0.16	2000								
Koroleva et al., 1986	S	Rat (Various)	4.6		-	5-100 V/cm2	0.9-3.5	-		10-100	5-10	-			10-40				BBB disruption.
Ballantine et al., 1960	S	Cat (Edinger-Westphal Nucleus)	2.7		-	1700	7.45		3	140	0.33			1-59			✓	✓	Lesions in 14 Cats (N=1-59) and not in 6 others (N=1-13).

Fry et al., 1958	S	Cat (LGN)												20-120		✓	×	No histologically observed legions.
Prieto et al., 2018	I	Cell cultures	43		-	50-90	1.71	-		200		-						
Kubanek et al., 2017	I	C. Elegans	10		5.5-15.4	11-30.6	0.6-1.0	200	20	0.5	1000	50						
Menz et al., 2017	I	Isolated Salamander Retina	43		-	0-120	0-1.98	-		1-1000		-						
	I	Isolated Salamander Retina	15		-	0-120	0-1.98	-		1-1000		-						
	I	Isolated Salamander Retina	0.5		-	1.6	0.23	-	2	100		-						
Kim et al., 2017	I	Hippocampal slice	0.5		-	780 uW/cm2	11.52 kPa	-		2.097 us	1.16 kHz	-		3 min				
Menz et al., 2013	I	Isolated salamander retina	43	30				1000	2		10 Hz - 1 MHz	50						Preliminary tests wirth additional high frequency modulation.
	I	Isolated salamander retina	43		-	0.06-60	0.04-1.4	-	2	1000	0.5	-						
Choi et al., 2013	I	Hippocampal neurons	0.5	16.1-92.8		19.6-122.4	0.8-2.0	5000	6	0.02	10-100	0.002-0.2		7x5 min				
Tyler et al., 2008	I	Hippocampal slice	0.44	0.023	-	2.9	0.31	-		22.7 us	0-100 (sweep)	-		5				LILFU-1.
	I	Hippocampal slice	0.44	0.023	-	2.9	0.31	-		22.7 us	10	-		0.5				Patch clamp recordings.
	I	Hippocampal slice, ex-vivo mouse brain	0.44	0.023	-	2.9	0.31	-		0.18	10	-		0.3				Transients.
	I	Hippocampal slice	0.44	0.023	-	2.9	0.31	-	8 min	22.7 us	0.002	-		36-48 h			×	Membrane integrity test, LILFU-1 every 8 min for 36-48 h.
	I	Hippocampal slice	0.67		-			-		74.5	10	-		0.57				Syanptic vesicle release.
Khraiche et al., 2008	I	Hippocampal slice	7.75		-	50-150 (I_TP)	1.28-2.21	-		1	2	-	2	60	0.55			
Bachtold et al., 1998	I	Hippocampal slice	0.5		-	140-110	1.89	-		100 ns	200 kHz	-		5 min				Assuming quoted ISPTA = ISPPA.
Rinaldi et al., 1991	I	Hippocampal slice	0.75		-	80	1.62	-		6 us	150 kHz	-		2-15 min				Assuming quoted ISPTA = ISPPA.

Study	Category	Organism	f (MHz)	I_SPTA (mW/cm2)	I_SPBA (W/cm2)	I_SPPA (W/cm2)	p (MPa)	BD (ms)	BI (s)	PL (ms)	PRF (Hz)	BDC (%)	TDC (%)	N	TL (s)	MI	Histology	Damage	Notes
Lee et al., 2014	H	Human (Fingertip)	0.35	3-100	0.003-0.1	0.3-10	0.118-1.148	10 s		0.1-1	10-100	1				0.1-0.97		×	
	H	Human (Fingertip)	0.65	3-100	0.003-0.1	0.3-10	0.21-1.145	10 s		0.1-1	10-100	1				0.13-0.71		×	
Legon et al., 2012	H	Human (Fingertip)	0.35		11.8	84.3	1.66	500	12	2	70	14		3x50					
	H	Human (Fingertip)	0.35		54.8	54.8	1.34	1000	12	10	100	100		3x50					
Dickey et al., 2012	H	Human (Fingertip)	1.117		-	0-410	3.66	-		100		-						×	
Gavrilov et al., 1977a	H	Human (Hand, forearm)	0.48		-	0-590	0-4.39	-		1-100		-						×	Pain thresholds
	H	Human (Hand, forearm)	0.887		-	0-8000	0-16.17	-		1-100		-						×	Pain thresholds
	H	Human (Hand, forearm)	2.67		-	0-30000	0-31.3	-		1-100		-						×	Pain thresholds
Downs et al., 2018	S	Mouse (Sciatic Nerve)	3.57		348-994	207-994	2.6-5.7	0.8-10.5			1000	35-100					✓	×	Successful parameters for EMG activity
	S	Mouse (Sciatic Nerve)	3.57		42.7-155	122-442	2-3.8	1			1000	35					✓	×	
	S	Mouse (Sciatic Nerve)	3.57		894	994	5.7	30			1000	90					✓	✓	
	S	Mouse (Sciatic Nerve)	3.57		803	892	5.4	500			1000	90					✓	✓	Positive control
Casella et al., 2017	S	Rat (Micturition Reflex)	0.25		2.1	28	0.95	300	2	0.252	2000				5-15 min				
Ni et al., 2016	S	Rat (Sciatic Nerve)	1	0.2											1 min/day				
Juan et al., 2014	S	Rat (Vagus Nerve)	1.1		13.6-93.4	74.8-102.8	1.83	15 s			200-1000	18.2-90.9				1.43-1.67			
Tych et al., 2013	S	Rat (Sciatic Nerve)	1.15		-	50-283	3.04	-		200		-							
Kim et al., 2012	S	Rat (Abducens Nerve)	0.35		4.6	8.6	0.53	200	1	0.36	1500	54		10			✓	×	8.6W/cm2 min for response.
	S	Rat (Abducens Nerve)	0.65		0.27-10.8	0.5-20	0.8	200	1	0.36	1500	54		10			✓	×	No responses.
Foley et al., 2008	S	Rat (Sciatic Nerve)	5.7		-	390	3.57	-		5 s		-					✓	×	
	S	Rat (Sciatic Nerve)	5.7		-	2255-7890	16.1	-		5 s		-					✓	✓	
Ellisman et al., 1987	S	Rat (Dorsal Nerve Roots)	2.4			1.9 (ISPTP=8.7)	0.53										✓	✓	Nerves in developing myelination stage
Gavrilov et al., 1977b	S	Cat (Pacinian Corpuscle)	0.48		-	0.4-2.5	0.11-0.29	-		0.1		-							
	S	Frog (Ear labyrinth)	0.48		-	0.01-1	0.02-0.18	-		1-100		-							
Lele et al., 1963	S	Earthworm (Giant Axon)	0.6		-	41	1.16	-	1	500		-					✓	✓	Damage for longer durations/intensities
	S	Earthworm (Giant Axon)	0.9		-	139.2	2.13	-	3	1400-2000		-					✓	✓	
	S	Earthworm (Giant Axon)	2.7		-	420	3.7	-		100-2000		-					✓	✓	
Young & Henneman, 1961	S	Cat (Saphenous Nerve)			-	700-800 V		-	2-3	400-900		-							
Wahab et al., 2012	S	Earthworm (Giant Axon)	0.825			0.1-15	0.1-0.7	15-75		50 us	100	0.5						✓	
Wright et al., 2017	I	Crab Leg Nerve Axon	0.67		10-84.5	20-169	1.8-2.4	8			10000	50			22 min			×	
	I	Crab Leg Nerve Axon	0.67		115-281	230-562	2.9-4.7	8			10000	50						✓	
	I	Crab Leg Nerve Axon	0.67		10-281	20-562	2.4-4.7	100			10000	50						✓	
	I	Crab Leg Nerve Axon	0.67		84.5	169	2.4-4.7	300		1	500	50						×	Lee et al., 2016 parameter replication
	I	Crab Leg Nerve Axon	1.1			17-475	0.7-4.2								22 min			×	No responses
	I	Crab Leg Nerve Axon	2			12-343	0.5-3.6								22 min			×	No responses
Colucci et al., 2009	I	Bullfrog (Sciatic Nerve)	0.661		-	100-575	1.81-4.33	-	1.5	30 s		-					✓	✓	Damage at higher intensities
	I	Bullfrog (Sciatic Nerve)	1.986		-	100-875	1.81-5.35	-	1.5	30 s		-							
	I	Bullfrog (Sciatic Nerve)	0.661					30 s	1.5	1-10	10-20								
	I	Bullfrog (Sciatic Nerve)	1.986					30 s	1.5	1-10	10-20								
Tsui et al., 2005	I	Bullfrog (Sciatic Nerve)	3.5		-	1-3 W		-		5 min		-							
Mihran et al., 1990	I	Frog (Sciatic Nerve)	2		-	0-40	0-1.14	-		0.5		-							
	I	Frog (Sciatic Nerve)	4		-	0-40	0-1.14	-		0.5		-							
Fry et al., 1950	I	Crayfish (Ventral Nerve)	0.98		35		1.07	2.8-43.5		0.5 - CW									
